# Synergizing Plasmonic Local Heating and 3D Nanostructures to Boost the Solar‐to‐Vapor Efficiency Beyond 100%

**DOI:** 10.1002/adma.202415655

**Published:** 2024-12-05

**Authors:** Pengfei Cheng, Malte Klingenhof, Hauke Honig, Lei Zhang, Peter Strasser, Peter Schaaf, Dangyuan Lei, Dong Wang

**Affiliations:** ^1^ Chair Materials for Electrical Engineering and Electronics Institute of Materials Science and Engineering and Institute of Micro and Nanotechnologies MacroNano TU Ilmenau Gustav‐Kirchhoff‐Str. 5 98693 Ilmenau Germany; ^2^ Department of Materials Science and Engineering, Department of Physics Centre for Functional Photonics Hong Kong Branch of National Precious Metals Material Engineering Research Centre, and Hong Kong Institute of Clean Energy City University of Hong Kong Kowloon Hong Kong 999077 China; ^3^ The Electrochemical Energy, Catalysis, and Materials Science Laboratory Department of Chemistry Chemical Engineering Division Technical University Berlin 10623 Berlin Germany; ^4^ Key Laboratory of Physical Electronics and Devices of Ministry of Education & Shaanxi Key Laboratory of Information Photonic Technique School of Electronic Science and Engineering Xi'an Jiaotong University Xi'an 710049 P. R. China

**Keywords:** 3D plasmonic sponge, nanostructured water–air interface, plasmonic local heating, solar water purification, solar‐to‐vapor efficiency

## Abstract

Solving the global challenge of freshwater scarcity is of great significance for over one billion people in the world. Solar water evaporation based on plasmonic nanostructures is one of the most promising technologies due to its high efficiency. However, the efficiency of this plasmonic nanostructure‐based technology can hardly achieve 100%. Therefore, it is highly desired to develop new solar converters utilizing plasmonic local heating and reasonable structure design to break the limit of solar‐to‐vapor efficiency for freshwater production. Here, a plasmonic sponge is developed as a solar evaporation converter with excellent full‐solar‐spectrum absorption, good heat localization performance, and fast evaporation kinetics through 3D nanostructures, achieving a 131% solar‐to‐vapor efficiency. Distinct from the traditional 2D localized heating‐based evaporation and nonmetallic 3D water evaporation, the 3D plasmonic sponge can simultaneously achieve highly efficient local heating and super large water–air interfaces for boosting solar‐to‐vapor efficiency. The 3D plasmonic sponge can be also used as a universal converter for purifying seawater, metal ion solutions, organic pollutant solutions, and strong acid and strong alkaline solutions. The full‐solar‐spectrum absorption, high efficiency, and universality in water purification suggest that the novel 3D plasmonic solar converter can bring a significant way to alleviate the crisis of freshwater resources.

## Graphical abstract

A self‐floating plasmonic sponge that harvests full‐solar‐spectrum light for highly efficient water purification.

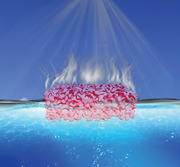

## 1 Introduction

Solar water purification is an environmentally friendly,^[^ ^1^, ^2^, ^3^ ^]^ and efficient technology to address the global energy scarcity and freshwater challenges.^[^ ^4^, ^5^, ^6^ ^]^ This technology can be applied to purify wastewater, seawater, and undrinkable water by evaporating water with solar converters.^[^ ^7^, ^8^, ^9^, ^10^ ^]^ Solar water evaporation works mainly in three schemes, namely bottom‐heating‐based evaporation (Figure S1a, Supporting Information), volumetric heating‐based evaporation (Figure S1b, Supporting Information), and interfacial heating‐based evaporation (Figure S1c, Supporting Information).^[^ ^11^, ^12^ ^]^ Due to the inevitable heat loss to the water and the environment, the solar‐to‐vapor efficiency of bottom‐heating‐based evaporation is relatively low (30–45%).^[^ ^11^ ^]^ Volumetric heating‐based evaporation can obtain a moderate efficiency (50–70%) by reducing surface heat loss and using optical nanofluids.^[^ ^13^ ^]^ By improving heat localization at the surface of the converter, interfacial heating‐based evaporation can achieve an efficiency up to >90%. However, the solar‐to‐vapor is still limited, since all three evaporation processes occur only at the 2D water–air interface with inevitable heat loss more or less. It is highly desired to boot up the solar‐to‐vapor efficiency even higher than 100% for practical applications.^[^ ^7^, ^9^, ^12^, ^14^, ^15^, ^16^ ^]^

Among the reported nanomaterials, metal nanomaterials supporting localized surface plasmon resonances (LSPRs) have recently attracted considerable attention in solar desalination.^[^ ^7^, ^10^, ^14^, ^16^, ^17^, ^18^, ^19^, ^20^ ^]^ The energy stored in plasmonic nanomaterials can be quickly released for local heating within 100 ps to 10 ns via a phonon‐phonon scattering process to keep the thermal equilibrium with the surrounding, enabling the effective photothermal conversion.^[^ ^21^, ^22^ ^]^ Most studies of plasmonic local heating focus on the efficiency enhancement under high solar intensity (>one sun), which requires expensive optical focusing elements and increases the installation and operating costs.^[^ ^10^, ^14^ ^]^ In contrast, nonmetal nanostructures have already demonstrated a solar‐to‐vapor efficiency of higher than 100% under one sun in very recent years.^[^ ^23^, ^24^, ^25^, ^26^ ^]^ However, plasmonic metal nanostructures that break the limit of solar‐to‐vapor efficiency under one sun while using a much cheaper preparation method and less expensive raw materials still remain great challenges.^[^ ^17^, ^27^, ^28^ ^]^ One possible strategy for achieving over 100% efficiency with metal nanomaterials is to realize a 3D water evaporation by combining localized heating‐based evaporation with 3D nanostructured interfaces. On one hand, localized heating‐based evaporation that occurs at water–air interface can offer a high evaporation efficiency through better thermal management, similar to interfacial heating‐based evaporation.^[^ ^11^, ^20^, ^29^ ^]^ On the other hand, confining water microscopically within 3D network channels by capillary force can significantly increase the area of water–air interfaces, which benefits solar‐to‐vapor efficiency.^[^ ^30^, ^31^, ^32^, ^33^, ^34^ ^]^ The 3D network channels have plenty of open pores and holes that can guide vapor out of the converter.^[^ ^8^, ^9^ ^]^ The real evaporation area (specific surface area) of the absorber is much larger than its projected top surface area (illumination area) of the converter. As the irradiation time increases, thermal energy diffused from the top to the bottom of the converter can be effectively utilized for evaporation in the 3D structures. The heat loss to the bulk water and environment is greatly reduced, which is inevitable in traditional converters. So the effective and efficient coupling between excellent thermal management and largely improved evaporation interface area can theoretically boost the solar‐to‐vapor efficiency exceeding 100%.^[^ ^8^ ^]^

Here, we develop a plasmonic sponge evaporator through in situ photodeposition of palladium nanoparticles (Pd NPs) onto a 3D nanostructured sponge, thereby achieving a solar‐to‐vapor efficiency reaching up to 131% under one sun. The plasmonic sponge demonstrates excellent broadband absorption (>97% across the wavelength range of 250–2500 nm) resulting from the multiple light scattering and the collective effect of plasmonic absorption of numerous Pd NPs hierarchically loaded onto the 3D sponge. This plasmonic sponge offers notable advantages over conventional solar evaporators, such as its versatility in seawater, metal ion solutions (including heavy metals), organic pollutant solutions, as well as strong acids and alkalis. The purified water from metal ion solutions and organic pollutant solutions presents comparable quality to de‐ion water. Additionally, the concentration of strongly acidic and strongly alkaline solutions decreases by 2–3 and 5–6 orders of magnitude, respectively. Moreover, it is lightweight, pressure‐resistant, and cost‐effective, and it can be easily synthesized on a large scale, which facilitates its commercialization. The proposed 3D plasmonic sponge for solar water evaporation will undoubtedly benefit areas that lack safe drinking water, which has long been recognized as a global challenge.

## 2 Results and Discussion

**Figure 1:**
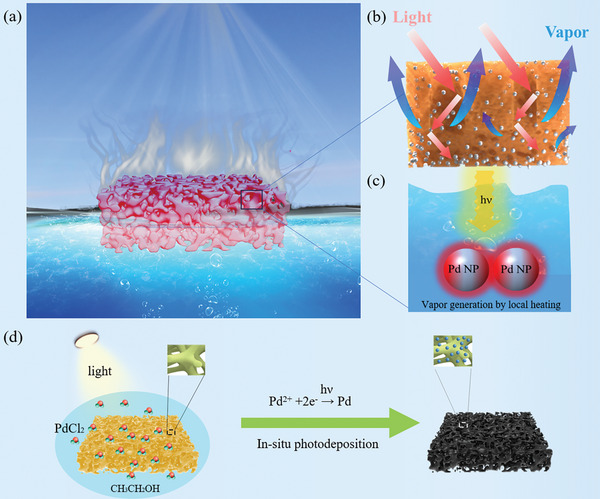
Schematics of the design and fabrication of a plasmonic sponge for efficient solar vapor generation. a) Schematic of plasmonic sponge‐based solar vapor generation. The water steam can be released easily from the surface of the porous plasmonic sponge under the solar light. b) Schematic of broadband light absorption induced by multiple scattering and collective plasmonic coupling among Pd NPs within the unique hierarchical nanostructure. c) Solar water evaporation is caused by local heating. d) The transition from a pristine sponge (yellow) to a plasmonic sponge (black) after undergoing the photodeposition of Pd NPs.

The plasmonic sponge is selected for efficient solar water evaporation due to four key reasons: 1) plasmonic nanostructures can offer super high and broadband absorption for local heating; 2) all‐in‐one structures, in which the light absorption layer also functions as the water transportation layer, ensure better efficient thermal management compared to double‐layer structures; 3) porous and superhydrophilic natures can supply enough water for evaporation; and 4) self‐floating property allows for large‐scale application. The black plasmonic sponge acts as an all‐in‐one self‐floating solar converter (**Figure** **1**a), which functions simultaneously as both an absorber and an evaporator with excellent thermal management, large evaporation area, and large water uptake because of the 3D structure. The plasmonic sponge demonstrates remarkable broadband light absorption, which is attributed to multiple light scatterings within its 3D structure and the collective plasmonic absorption effect of numerous Pd NPs (Figure 1b).^[^ ^35^, ^36^ ^]^ The Pd NPs attached to the micro/nanochannels serve as localized heating sources due to the localized surface plasmon resonances (LSPRs) and their coupling effect (Figure 1b,c). The strong local heating can be released within 100 nanoseconds (ns) and quickly induces water evaporation (Figure 1c).^[^ ^37^, ^38^ ^]^ The low thermal conductivity of 3D micro/nanochannels and localized heating behavior enable the converted thermal energy to be utilized more efficiently for evaporation, while heat loss to the bulk water and the environment can be minimized. To achieve high efficiency of solar water evaporation, it is also essential to have a large evaporation area, great water uptake,^[^ ^39^ ^]^ self‐floating behavior, which can be well satisfied by the large quantities of crisscrossed micro/nanochannels of the lightweight plasmonic sponge.

In contrast to the complex and expensive fabrication methods required for traditional plasmonic metal nanostructures for solar water evaporation, such as costly anodized aluminum oxide (AAO) templates and physical vapor deposition (PVD) equipment (**Table** **1**), we developed a plasmonic sponge synthesized by repeatedly depositing Pd NPs onto a sponge using a simple and environmentally friendly photodeposition process of PdCl_2_ in an ethanol solution. (Refer to the experimental section for more details). The reaction energy source is from a solar light simulator. The whole synthetic process does not require any other chemicals and expensive equipment (Table S1, Supporting Information). After the photodeposition of Pd NPs (Figure 1d), the pristine yellow sponge transformed into a completely black sponge, allowing for highly efficient light absorption for solar water evaporation.

**Table 1:** Solar water evaporation performance of the plasmonic sponge compared with the traditional plasmonic‐based interfacial evaporation.^[^ ^7^, ^10^, ^14^, ^15^, ^16^, ^17^, ^37^, ^48^ ^]^

Materials	Method	Evaporation rate [kg m^−2^ h^−1^]	Absorption range [nm]	Average absorptance	Solar‐to‐vapor efficiency	Condition	Refs.
Nanoporous Au	Dealloying	1.51	300–2500	86%	94.5%	1 sun	[7]
3D Al NPs/AAO^a^	PVD + AAO^c^	≈7.7	400–2500	96%	91%	6 suns	[10]
3D Au NPs/AAO	PVD + AAO	≈0.8	400–2500	99%	54%	1 sun	[14]
Carbon sponge	Carbonization	1.39	250–2500	96%	90%	1 sun	[15]
Pd/wood	Wet chemistry	≈1	250–2500	99%	85%	10 suns	[16]
Black Au	PVD + AAO	15.59	400–2500	91%	57%	20 suns	[37]
Au/NPT^b^	PVD + AAO	1.202	250–2500	≈95%	80.5%	1 sun	[48]
Nanoporous Cu	Dealloying	7.47	200–2500	≈90%	93.7%	5 suns	[17]
Pd/sponge	photodeposition	2.022	250–2500	97%	131%	1 sun	This work

Note: AAO: Anode aluminum oxide, NPT: Nanoporous templates, PVD: physical vapor deposition.

**Figure 2:**
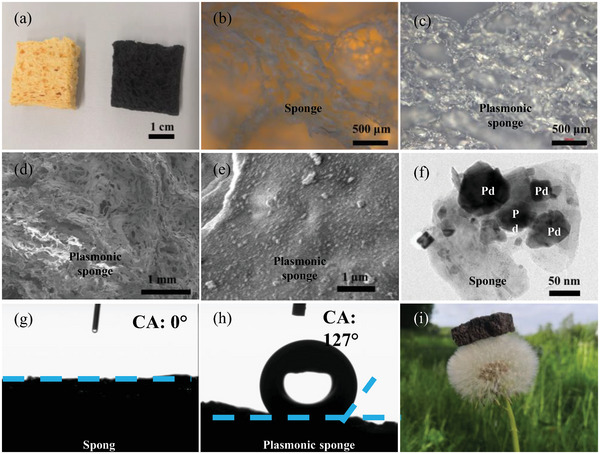
Characterizations of the plasmonic sponge. a) Optical photograph of a pristine sponge (left) and a plasmonic sponge (right). Optical microscopy (OM) images of b) the pristine sponge and c) the plasmonic sponge. d) Low‐ and e) high‐magnification SEM images of the plasmonic sponge. f) TEM image of the plasmonic sponge. CA measurements on g) the pristine sponge and h) the plasmonic sponge. i) The lightweight plasmonic sponge on a dandelion.

It is a very feasible method to modify the optical properties of porous structures using plasmonic metallic NPs, such as Pd NPs. In our case, the originally yellow sponges changed to a completely black color (**Figures** **2**a and S2a–d, Supporting Information) after the photodeposition, directly demonstrating their broadband light absorption capabilities. The plasmonic sponge can be easily shaped into different forms (Figure S2b–d, Supporting Information), highlighting its excellent processability. In addition, this method can be extended to upload the NPs on various porous materials, such as nickel foam and dust‐free cloth (Figure S2e–h, Supporting Information), as confirmed by X‐ray diffraction (XRD) results (Figure S3a–c, Supporting Information). It is noted that the pristine sponge already possessed numerous hole structures (Figure 2a). These hole structures are still retained after the photodeposition process, which benefits both light trapping and vapor release during evaporation.

Figure 2b,c present the optical microscopy (OM) images of the thin pristine sponge and the plasmonic sponge, respectively. The pristine sponge shows abundant pore structures and light can easily penetrate them. While light is greatly trapped within the plasmonic sponge due to the LSPR effect and collective plasmonic effect. The pristine sponge has a hierarchical porous structure, ranging from micrometer‐scale porous (Figure 2b–d) to nanometer‐scale porous (Figure S4, Supporting Information). The plasmonic sponge inherits a similar porous structure from the pristine sponge. The open pore structures of plasmonic sponges are further investigated by scanning electron microscopy (SEM) and transmission electron microscopy (TEM) characterizations (Figure 2d–f). Both SEM (Figure 2e) and TEM (dark area in Figure 2f) images reveal that Pd NPs, with a means size of 40 nm (Figure S5, Supporting Information), were closely attached to the sponge. For comparison, the Pd NPs were also deposited on a silicon substrate. The Pd NPs on the sponge via in‐situ deposition ensures the perfect contact between Pd NPs and the sponge surface, which is directly capable of heating up water to vapor (Figure 1d) and thus improving the evaporation rate.

The contact angle (CA) measurement confirms that the pristine sponge in the dry state (without water uptake) is hydrophilic with a CA close to zero (Figure 2g). The outstanding hydrophilicity of a pristine sponge is beneficial for water self‐pumping to the top of the sponge. In contrast, after decorating with Pd NPs, the plasmonic sponge in a dry state exhibits hydrophobic characteristics with a large CA of 127° (Figure 2h). The water uptake capacity is primarily determined by the porous structure. However, for the hydrophobic plasmonic sponge, it is necessary to manually press the sponge to facilitate water uptake. When the plasmonic sponge is released from the hand and its shape is restored due to its excellent elastic deformation property (able to withstand up to 20% compression strain, Figure S6, Supporting Information), water is effectively pumped into the porous structure. During evaporation, water can be continuously self‐pumped into the porous sponge to maintain the pressure balance by capillary force. As a result, the plasmonic sponge in a wet state turns to a superhydrophilic property (Video S1, Supporting Information). Its maximum weight of water uptake can be as high as more than 13 times compared with its initial weight (Figure S7, Supporting Information). The saturated density (0.686 g cm^−3^) of wet plasmonic sponge has increased over nine times (Figure S8, Supporting Information), but remains smaller than that (1.0 g cm^−3^) of water, allowing the plasmonic sponge to naturally float on water (Figure S9, Supporting Information). The super lightweight nature of the 3D plasmonic sponge can be confirmed by freely standing on a dandelion flower without clear deflection (Figure 2k). Notably, the plasmonic sponge is able to withstand a 2 kg of water bottle, and no significant mechanical damage is observed (Figure S10, Supporting Information), demonstrating its excellent pressure resistance. Therefore, the self‐floating behavior, super lightweight nature, and pressure resistance of plasmonic sponge make it highly advantageous for practical applications.

Furthermore, the specific surface area of the plasmonic sponge has been determined using Brunauer–Emmett–Teller (BET) measurement technique (Figure S11, Supporting Information). The maximum evaporation interface area for the plasmonic sponge (size: 3.0 × 3.0 × 1.0 cm^3^) can achieve as high as 5981 cm^2^, which is orders of magnitude larger than its projected surface area (illumination area) of only 9 cm^2^. Clearly, the 3D microstructured water–air interface with large specific area is very helpful to improve the evaporation efficiency.

The as‐prepared plasmonic sponge demonstrates exceptional light‐trapping performance and solar‐thermal (photothermal) conversion capabilities. UV–Vis–NIR reflection and absorption measurements are carried out to quantitatively investigate the broadband absorption property of the pristine sponge and plasmonic sponge. The pristine sponge exhibits a large part of reflection in the wavelength range from 250 to 2500 nm (Figure S12, Supporting Information), which covers the entire solar spectrum, while the plasmonic sponge shows a very low reflection in the same spectral range. Furthermore, it is observed that the wet plasmonic sponge exhibits significantly lower reflection in this spectral range than the dry one, likely due to the higher refractive index of water (*n* = 1.33) than the air (*n* = 1).^[^ ^40^ ^]^ The absorption can be calculated by the following equation

**Figure 3:**
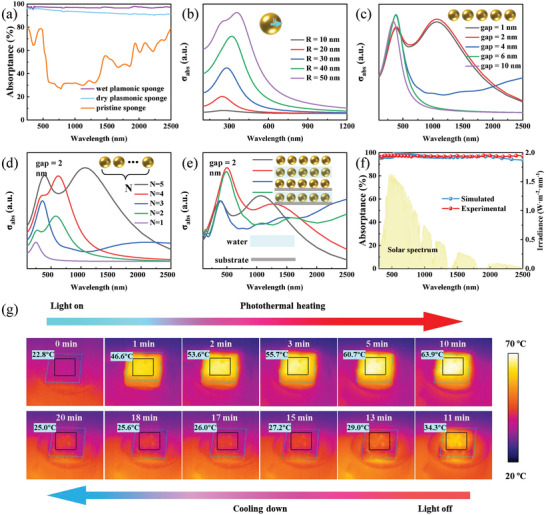
Broadband absorption and photothermal properties of the plasmonic sponge. a) Experimental absorption spectra of pristine sponge, dry plasmonic sponge, and wet plasmonic sponge. Dependent of simulated absorption cross section on b) size, c) gap distance, d) hybridization degree and e) dielectric environment (in air, in water, on the sponge and in water + on the sponge). f) Experimental and simulated absorption of plasmonic sponges. The light‐yellow color shows the AM 1.5G solar spectrum. g) Photothermal images of the plasmonic sponge under one sun illumination and without light illumination.

(1) A=1−R−T

where A, R, and T stand for absorptance, reflectance, and transmittance, respectively. In our case, T equals zero, while R refers to the measured data. The wet plasmonic sponge achieves very high broadband absorption, averaging up to 97% across the entire solar spectrum, which is much higher than the counterpart of the pristine sponge (**Figure** **3**a). In addition, Raman measurements further verify its excellent light‐trapping performance, because no Raman signal is detectable due to the near‐unity absorption of the plasmonic sponge absorber (Figure S13, Supporting Information).

In order to investigate the plasmonic effects on excellent broadband absorption in detail, finite‐difference time‐domain (FDTD) simulations were conducted. As the radius increases, the absorption of an individual Pd NP is significantly enhanced, and the absorption redshifts (Figure 3b). As experimentally observed, Pd NPs are often close to each other. Therefore, The coupling effect of Pd NPs was simulated by varying the spacing gaps. The absorption can be remarkably enhanced due to the strong coupling effect with a gap size of 1 or 2 nm (Figure 3c), which is also applied to another size of Pd nanoparticles (Figure S14, Supporting Information). The absorption band can be further broadened by increasing the number of Pd NPs close to each other (Figure 3d). Besides, during evaporation, the surrounding medium of Pd NPs is water and sponge, and the different dielectric environments will also result in absorption variation (Figure 3e).^[^ ^19^, ^36^, ^41^ ^]^ In addition to the factors above, it is particularly important to study the effect of multiple scattering and the collective effect of many NPs distributed in 3D structures on enhanced broadband absorption. Therefore, an FDTD simulation is performed, where 1000 Pd NPs with diameters ranging from 10 to 60 nm are randomly distributed in a 3D space (*x* × *y* × *z* = 200 × 200 × 1050 nm^3^). The simulated absorption spectrum almost perfectly matches the measured result (Figure 3f). The localized plasmonic mode of a single particle and the coupling effect can significantly enhance the local field (Figure S15, Supporting Information), which will lead to a localized heating effect and better thermal management via a non‐radiative decay process.

The development of converters with heat localization and better thermal management is crucial for achieving high efficiency of solar water evaporation.^[^ ^12^, ^20^, ^38^, ^42^ ^]^ Our plasmonic sponge also exhibits excellent heat localization properties, thanks to the presence of plasmonic Pd NPs acting as local heating sources. Generally, when the frequency of the incident excitation wavelength matches the oscillation frequency of free electrons in metal nanostructures, a strong coherent resonance between the photons and electrons triggers collective electron oscillations, leading to the generation of plasmons (Figure S16, Supporting Information). Then, a plasmon quantum is transformed into a pair of hot electrons and the hot hole through Landau damping, occurring on a timescale of 1 to 100 fs. Subsequently, the excited‐state hot electrons transfer their energy to lower‐energy electrons in a process of electron‐electron scattering, within a timeframe of 100 fs to 1 ps. The remaining hot carriers then continue to transfer energy to the plasmonic metal nanoparticle through electron‐phonon scattering over a period of 1–100 ps. Finally, the plasmonic nanoparticle establishes thermal equilibrium with its surroundings through a phonon–phonon interaction, and the heat release process is called local heating,^[^ ^21^ ^]^ which is an ultrafast process and therefore ensures highly efficient solar‐thermal conversion. As is presented in Figure S17 (Supporting Information), the dependence of the surface temperature versus on time is plotted under the solar intensity of 100 mW cm^−2^ (AM 1.5G). In the beginning, the plasmonic sponge demonstrates a surface temperature of 22.8 °C when no illumination. However, upon illumination, its surface temperature rapidly rises to 53.6 °C within the first 2 min due to heat localization. Subsequently, it gradually increases to 63.9 °C after 10 min of continuous illumination (Figure 3g). However, once the light is turned off, the surface temperature rapidly decreases back to 34.3 °C within 1 min, indicating the loss of heat. Therefore, continuous irradiation‐induced local heating can greatly boost liquid‐vapor phase change.

**Figure 4:**
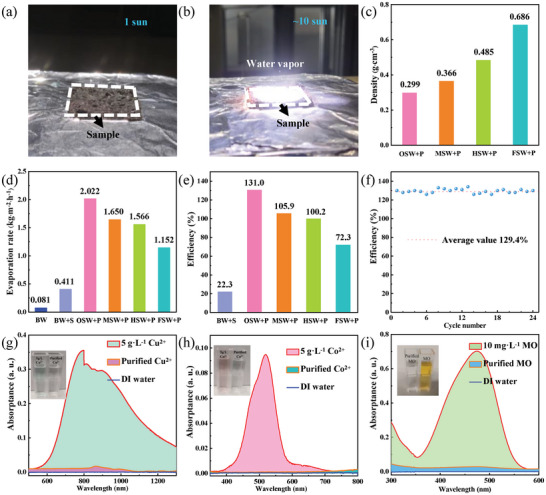
Solar vapor generation performance using the plasmonic sponge. Optical photographs of water evaporation under a) one‐sun and b) 10‐sun illumination. c) Four wet plasmonic sponges with different densities. d) Photothermal water evaporation rates of the four samples in (c), compared with bulk water (BW) and BW in the pristine sponge (BW+S). e) Solar‐to‐vapor efficiency of the six samples in (d). f) Stability tests for the plasmonic sponge (OSW+P). g–i) UV–Vis–NIR absorption spectra of g) Cu^2+^, h) Co^2+^, and i) MO solutions before and after purification by the plasmonic sponge. The insets are the corresponding photographs before and after purification.

The solar water evaporation performance of a 3D plasmonic sponge is systematically explored in **Figure** **4**. Under the illumination of one sun (100 mW cm^−2^), almost no vapor (Figure 4a) can be observed, while a significant amount of vapor (Figure 4b; Video S2, Supporting Information) is generated under the illumination of ∼ ten suns, suggesting a faster evaporation rate at higher solar intensity. By increasing the amount of soaked water (Figure 4c), the water–air interface area can be regulated accordingly due to the 3D structure and superhydrophilic nature of the plasmonic sponge (Video S1, Supporting Information). The plasmonic sponges that soaked different amounts of water are referred as FSW+P (0.686 g cm^−3^), HSW+P (0.485 g cm^−3^), MSW+P (0.366 g cm^−3^) and OSW+P (0.299 g cm^−3^), which represent fully soaked water, highly soaked water, moderately soaked water and optimally soaked water in plasmonic sponges (Figure S18, Supporting Information), respectively. The evaporation rates of these samples were measured under one sun. For comparison, the evaporation rates of bulk water (BW) without sponge and bulk water in the pristine sponge (BW+S) were also measured under the same conditions. The evaporation rates of BW, BW+S, OSW+P, MSW+P, HSW+P, and FSW+P are 0.081, 0.411, 2.022, 1.650, 1.566, and 1.152 kg m^−2^ h^−1^, respectively (Figure 4d).

The energy conversion efficiency of solar‐to‐vapor^[^ ^8^, ^30^ ^]^ can be expressed as^[^ ^30^, ^43^ ^]^

(2) $$\eta =\frac{r\Delta H}{Q}\;$$

where *η* is the solar‐to‐vapor efficiency; *r* is the evaporation rate (*r* = *r*_light_ – *r*_dark_); Δ*H* is the evaporation enthalpy of water and *Q* is the incident light energy density. In the extreme case that *η* = 100%, *Q* = 1 kWh m^−2^ and Δ*H* = 2.43 × 10^6^ J kg at 30 °C, then *r* is calculated to be 1.481 kg m^−2^ h^−1^.^[^ ^8^, ^30^ ^]^ Obviously, the highest evaporation rate of 2.022 kg m^−2^ h^−1^ achieved by OSW+P sample greatly exceeds the theoretical limit. According to the Kelvin equation,^[^ ^44^ ^]^

(3) $$ln\;\;\frac{P}{P_{0}}=\frac{2\gamma V}{\rho R^{\prime }T^{\prime }}\;$$

where *P*, *P*_0_, *γ*, *V*, ρ, *R*, ***^’^*** and *T* ***^’^*** represent the actual vapor pressure, saturated vapor pressure, surface tension, molar volume, radius of liquid drop, gas constant, and absolute temperature, respectively. When the shape of the liquid changes from a flat surface to a nanostructured one (Figure S19, Supporting Information), the ρ becomes much smaller. As a result, the ratio of the actual vapor pressure *P* to the saturated vapor pressure *P*_0_ increases, which can remarkably promote water evaporation. Furthermore, the evaporation enthalpy of water within the range from 30 to 40 °C (Figure S20, Supporting Information) only decreases slightly, which can be considered unchanged in our interfacial evaporation system.^[^ ^16^, ^44^ ^]^ The exceptional performance characteristics can be attributed to the synergetic effect of the 3D plasmonic sponge, which offers almost perfect broadband absorption, extremely large area of water–air interface, excellent thermal management with heat localization, and abundant water uptake through the 3D nanostructures.

In the FSW+P sample, the porous structure is completely filled with water (Figure S18a, Supporting Information), and the evaporation process can be considered to occur solely at the 2D water–air interface, achieving an efficiency of ≈72.3% (by using Equation 2) mainly due to the localized heating effect (Figure 4e). The situation is quite different in the case of the OSW+P sample (Figure S18d, Supporting Information), where an optimal amount of water is partially soaked in the plasmonic sponge, creating a large specific area of water–air interface in the microchannels and open pores of the 3D structure. This results in a total efficiency of 131% (Figure 4e), which is clearly superior than the most recent work conducted under one sun (Table 1; Table S2, Supporting Information). It should be noticed that the obtained efficiency clearly beyond 100% is mainly due to the calculation in Equation (2) using the value of the standard evaporation enthalpy of water without any assistance of evaporators. With the help of the evaporator (such as due to the Kelvin effect Equation (3) and larger specific surface area for evaporation), the real value of evaporation enthalpy of the converter system can be clearly reduced, but it is not suitable for the purpose of performance comparison between different evaporators. Therefore, it is well‐accepted using the value of the standard evaporation enthalpy of water for the calculation and for better comparison.^[^ ^27^, ^45^, ^46^, ^47^ ^]^

Moreover, the solar‐to‐vapor efficiency of the plasmonic sponge does not show any significant decline after 24 cycles of photothermal water evaporation experiments (Figure 4f). The excellent structural and optical stabilities of the plasmonic sponge are also confirmed by Figures S21–S23 (Supporting Information). It also remains stable solar‐to‐vapor efficiency under the simulated seawater (Figure S24a, Supporting Information), but its efficiency (Figure S24b, Supporting Information) shows an apparent decrease as the times of evaporation, probably because the cellulose sponge is not acid resistant. We also conducted outdoor evaporation tests. The outdoor solar‐to‐vapor efficiency is slightly higher than in the laboratory (Figure S25, Supporting Information), which might be caused by airflow (wind velocity: 3–4 m s^−1^) during the evaporation period. In the outdoor stability test, the solar‐to‐vapor efficiency of the plasmonic sponge indicates remarkable stability, maintaining consistent performance even after four cycles of evaporation tests.

Significantly, our plasmonic sponge also performs exceptionally well in water purification (experimental setup shown in Figure S26, Supporting Information), particularly with the (heavy) metal ion‐contained solution, organic pollutant solution, seawater, acid solution, and alkaline solution. As demonstrated in Figures 4g,h and S27–S29 (Supporting Information), when we used 5 g L^−1^ CuCl_2_ solution (a typical heavy metal ion‐contained solution), 5 g L^−1^ CoCl_2_ solution and 5 g L^−1^ Ni(NO)_2_ solution (two kinds of metal ion‐contained solution) as water sources for evaporation, there were almost no metal ion signals detected in the UV–vis absorption spectra. After evaporation, the colored CuCl_2_ solution (light blue), CoCl_2_ solution (light pink), and Ni(NO)_2_ solution (light green) changed into completely transparent (Figure S28, Supporting Information), demonstrating that high‐quality water can be obtained using solar vapor generation for dealing with nonferrous metal ion‐contained solutions. The precise concentrations of Cu^2+^, Co^2+,^ and Ni^2+^ are detected by the ion‐coupled plasma‐optical emission spectroscopy (ICP‐OES), showing a decrease in concentration by about three orders of magnification compared to the pristine sample (Figure S29, Supporting Information). We further explored this technology for organic pollutant solution treatment. Methyl orange (MO) solution is one of the most common but harmful organic pollutants to the environment.^[^ ^49^, ^50^ ^]^ After the solar vapor generation experiment, no MO signal for the treated MO solution was detected (Figure 4i). In addition, its absorption spectrum is close to pure deionized (DI) water, indicating the resultant solution contained negligible amounts of MO. The performance of seawater desalination was also investigated. The results demonstrated that the purified seawater can satisfy the standard of the World Health Organization (WHO) (Figure S30, Supporting Information). Furthermore, we explored the applicability of our photothermal water evaporation technology with strong acid (0.5 m H_2_SO_4_) and strong alkaline (1 m NaOH) solutions. The results revealed a significant decrease in the concentrations of H^+^ and OH^−^ by 2–3 orders of magnitude and 5–6 orders of magnitude, respectively (Figure 4k–l). This corresponded to a shift in the pH values of 0.5 M H_2_SO_4_ from 0 to 2–3 (Figure S31a, Supporting Information) and of 1 m NaOH 14 from 8 to 9 (Figure S31b, Supporting Information), respectively, verifying the feasibility of this technology for effectively treating strong acid and strong alkaline solutions.

We have developed a novel 3D convertor of plasmonic sponge to allow water only confined in nano/microchannels and promote solar‐to‐vapor efficiency beyond the theoretical limit. The high performance of the plasmonic sponge stems from several key features: 1) plasmonic nanostructures can offer high and broadband absorption for local heating; 2) all‐in‐one structures, in which the light absorption layer also functions as the water transportation layer, ensure better efficient thermal management compared to double‐layer structures; and 3) porous and superhydrophilic structures can supply enough water for evaporation. Together, these features synergistically improve the solar‐to‐vapor efficiency.

## 3 Conclusion

A 3D plasmonic sponge is developed as a solar water purification converter with water evaporation efficiency clearly exceeding 100% and excellent purification performance. The plasmonic sponge offers several key advantages, including super‐high broadband absorption, large specific surface area of water–air interface, outstanding thermal management with localized heating, and large water uptake. It is clear that the synergetic effect of all these advantages is the reason for its excellent performance. Compared to the conventional evaporation at 2D water surface, such as bottom‐heating‐based evaporation, volumetric heating‐based evaporation, and interfacial heating‐based evaporation, the critical advantages of the 3D plasmonic sponge rely on both evaporation at microchannels and open pores and heat localization via Pd NPs throughout the whole 3D structure. In addition, this method can be universally applied to treat seawater, (heavy) metal ion solutions, organic pollutant solutions, strong acid and strong alkaline solutions, demonstrating its versatility for obtaining freshwater. In the purified water from metal ion solutions and organic pollutant solutions, harmful substances can be barely detectable, and the concentration of strong acid and strong alkaline solutions can be reduced by 2–3 and 5–6 orders of magnitude. Furthermore, its facile and low‐cost fabrication, ultra‐lightweight, self‐floating behavior, and excellent stability indicate its great potential for practical applications. We believe that the developed plasmonic sponge provides a very promising way to alleviate the crisis of freshwater resources.

## 4 Experimental Section

### Materials and Chemicals

The sponge was purchased from Greenet Company. Palladium chloride (PdCl_2_) and ethanol were bought from Sigma‐Aldrich.

### Synthesis of Pd NPs in Sponge

The sponge was immersed in a bottle with 25 mL ethanol. Then, ≈20 mg PdCl_2_ powder was dissolved in ethanol. Finally, the bottle was sealed by a parafilm and was stirred on the stirring stage. The distance between the bottle and light source (LOT, Quantum Design GmbH) was kept ≈10 cm. The sponge was turned upside down at every interval of 30 min to make sure the Pd NPs could be uniformly distributed in it. In addition, ≈20 mg PdCl_2_ powder was added at every interval of one hour. This process was repeated seven times to form a totally black sponge. The energy source in the whole reaction process was light energy without any other additional sources.

### Characterizations

Optical microscopy (OM, Zeiss), SEM (Hitachi S‐4800) were used to obtain the structure image of prepared samples. TEM (JEOL JEM 2100) and probe‐Cs corrected (JEM‐ARM300F2 cold FEG with JEOL dual‐SDD EDS detector (2 × 158 mm^2^) were applied to investigate the surface morphology and particle distribution. The wettability test was conducted by a CA machine (Krüss DSA 10 Mk2). The optical spectrum response of the samples was measured by a UV–VIS–NIR (from 230 to 2500 nm) spectrophotometer (Cary 5000) with an integrating sphere and it was also applied to measure the quality of purified water. The infrared (IR) images were recorded by an IR camera (FLIR SYSTEM, Thermovision A40) to investigate the temperature distribution. BET measurement was tested by the Anton Paar Nova 800. Mechanical tests are conducted by Hegewald & Peschke, Inspekt table 20 kN with 500 N force measurement cell. ICP‐OES was used to detect the concentrations of metal ion in seawater and of (heavy) metal ion solutions.

### Simulation

A commercial FDTD software (Ansys/Lumerical Company) was used to study the interaction of light and Pd NPs. The interaction of light and Pd NP was investigated by performing absorption, scattering, and extinction cross‐section calculations.^[^ ^35^ ^]^ A total field scattering field (TFSF) source with a wavelength from 100 to 2000 nm was applied in the simulation. A perfectly match layer (PML) was used along *z*‐axis, while symmetric and anti‐symmetric boundary conditions were applied along *x*‐ and *y*‐axes. The dielectric functions of Pd were obtained from the experiment.^[^ ^51^ ^]^ The refractive index of water, ethanol, and cellulose sponge are 1.33, 1.36, and 1.47, respectively.^[^ ^40^, ^52^ ^]^

### Photothermal Water Evaporation Experiments

All the solar desalination experiments were carried out under a solar simulator (LOT, Quantum Design GmbH). The solar intensity was adjusted by a certified photodiode and an optical power meter (Newport, 1916‐R, with a detector, 818‐UV). The samples were placed in de‐ion water, seawater, MO solution or (heavy) metal ion solutions for solar steam generation. For water evaporation experiments, a shelter was used to keep the illumination area of 3 × 3 cm^2^ (Figure 4a,b), while for water purification experiments, the experimental set‐up (Figure S26, Supporting Information) was used to collect the purified water. A high‐precision electronic balance (KERN, precision: 0.1 mg) was applied to record the mass differences (Δm). The surface temperature of the plasmonic sponge was recorded in real time by an IR camera and a thermometer.

## Conflict of Interest

The authors declare no conflict of interest.

## Supporting information



Supporting Information

Supplemental Video 1

Supplemental Video 2

## Data Availability

The data that support the findings of this study are available from the corresponding author upon reasonable request.
