# Supplementary material for: Synergizing Plasmonic Local Heating and 3D Nanostructures to Boost the Solar‐to‐Vapor Efficiency Beyond 100%
Source: Adv Mater. 2024 Dec 5;37(5):2415655. doi: 10.1002/adma.202415655 (PMC11795728; doi:10.1002/adma.202415655)
Supplement: Supplementary file 1 — Supporting Information [file ADMA-37-2415655-s001.docx]

**Supporting Information**

Synergizing Plasmonic Local Heating and 3D Nanostructures to Boost the Solar-to-Vapor Efficiency beyond 100%

*Pengfei Cheng*,^1,2^ *Malte Klingenhof*,^3^ *Hauke Honig,^1^ Lei Zhang,^4^ Peter Strasser*,^3^ *Peter Schaaf*,^1^ *Dangyuan Lei*,^2*^ *Dong Wang*^1^*

^1^ Chair Materials for Electrical Engineering and Electronics, Institute of Materials Science and Engineering and Institute of Micro and Nanotechnologies MacroNano, TU Ilmenau, Gustav-Kirchhoff-Str. 5, 98693 Ilmenau, Germany

^2^ Department of Materials Science and Engineering, Centre for Functional Photonics, and Hong Kong Branch of National Precious Metals Material Engineering Research Centre, City University of Hong Kong, Kowloon, Hong Kong 999077, China

^3^ The Electrochemical Energy, Catalysis, and Materials Science Laboratory, Department of Chemistry, Chemical Engineering Division, Technical University Berlin, 10623 Berlin, Germany

^4^ Key Laboratory of Physical Electronics and Devices of Ministry of Education & Shaanxi Key Laboratory of Information Photonic Technique, School of Electronic Science and Engineering, Xi'an Jiaotong University, Xi'an 710049, P.R. China

* Corresponding authors: dong.wang@tu-ilmenau.de; dangylei@cityu.edu.hk


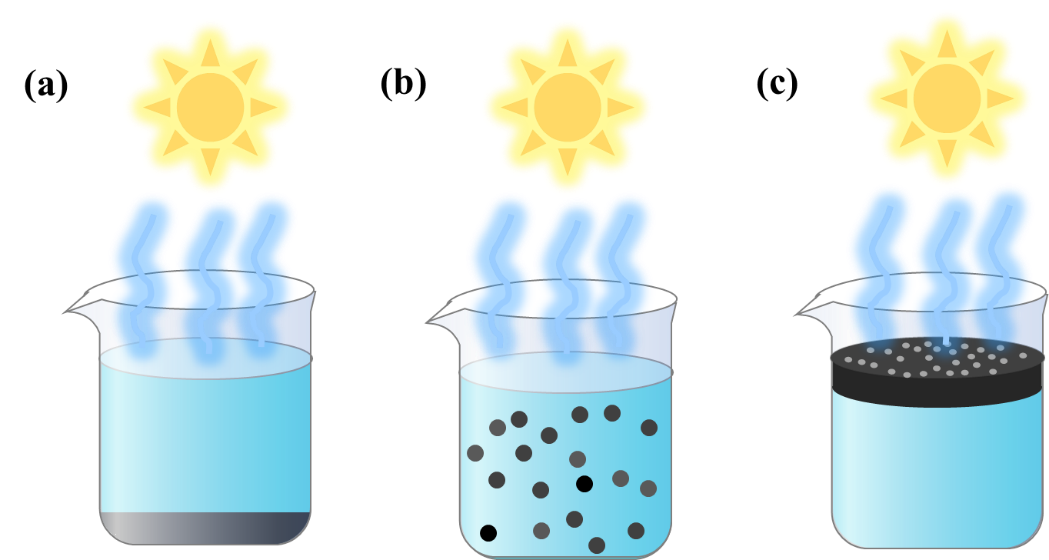


**Figure S1** (a) Bottom-heating-based evaporation. (b) Volumetric heating-based evaporation. (c) Interfacial heating-based evaporation.


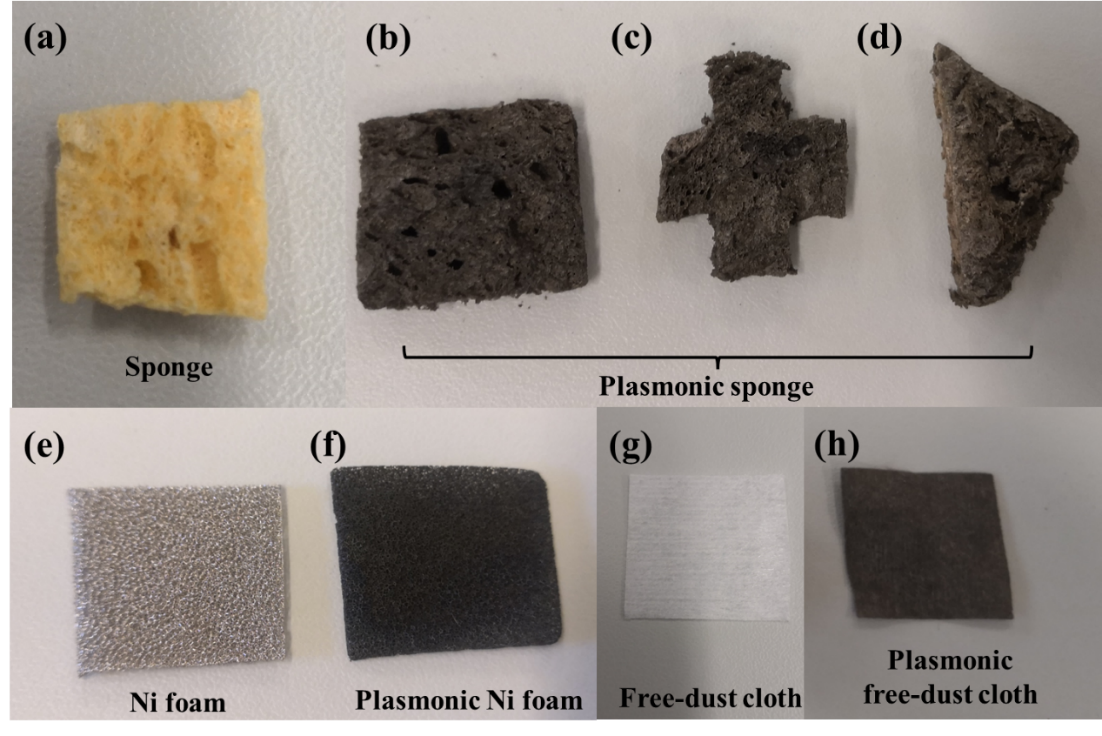


**Figure S2** (a) Photographs of sponges. Different shapes of plasmonic sponge (b) rectangle, (c) cross and (d) triangle. (e) Nickel (Ni) foam. (f) Plasmonic Ni foam. (g) dust-free cloth. (h) Plasmonic dust-free cloth.

**
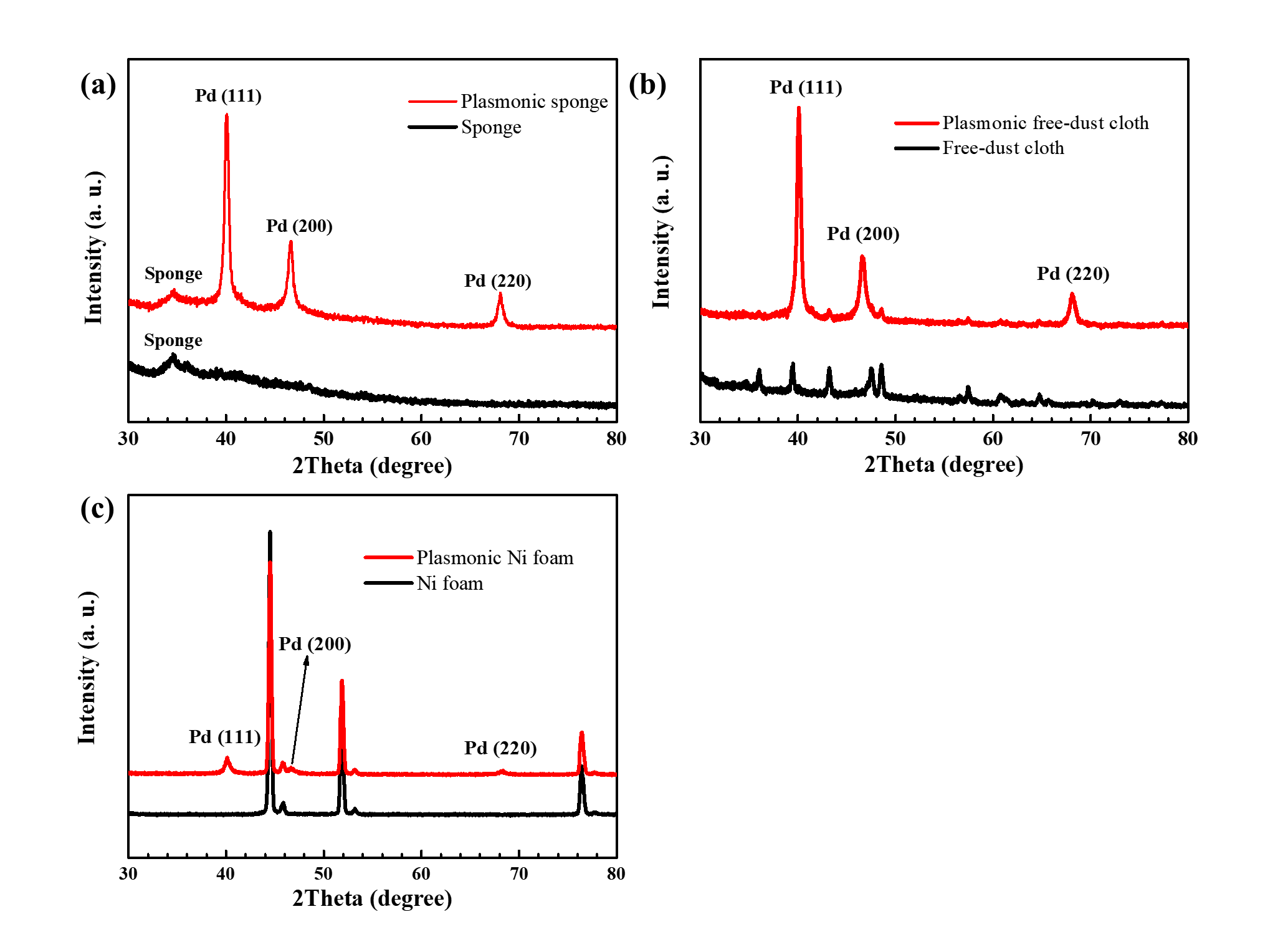
**

**Figure S3** XRD pattern of (a) sponge and plasmonic sponge, (b) Ni foam and plasmonic Ni foam and (c) dust-free cloth and plasmonic dust-free cloth.


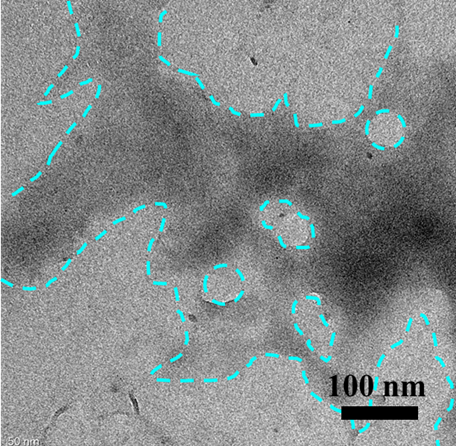


**Figure S4** TEM image of the sponge used for photodeposition. The dash lines were applied to show the nanostructures of sponge.


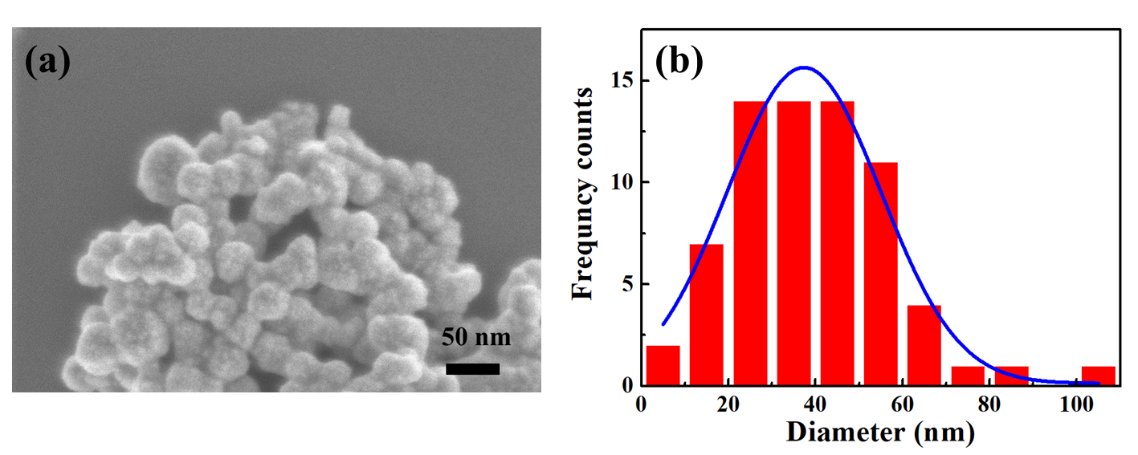


**Figure S5** (a) SEM image of Pd nanoparticles deposited without the sponge as the support. (b) The corresponding size distribution in (a).


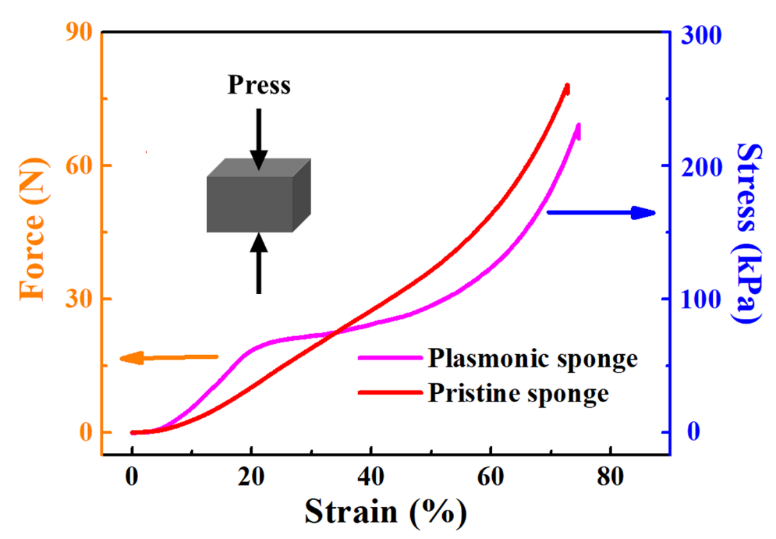


**Figure S6** Mechanical property test for a plasmonic sponge and a pristine sponge.


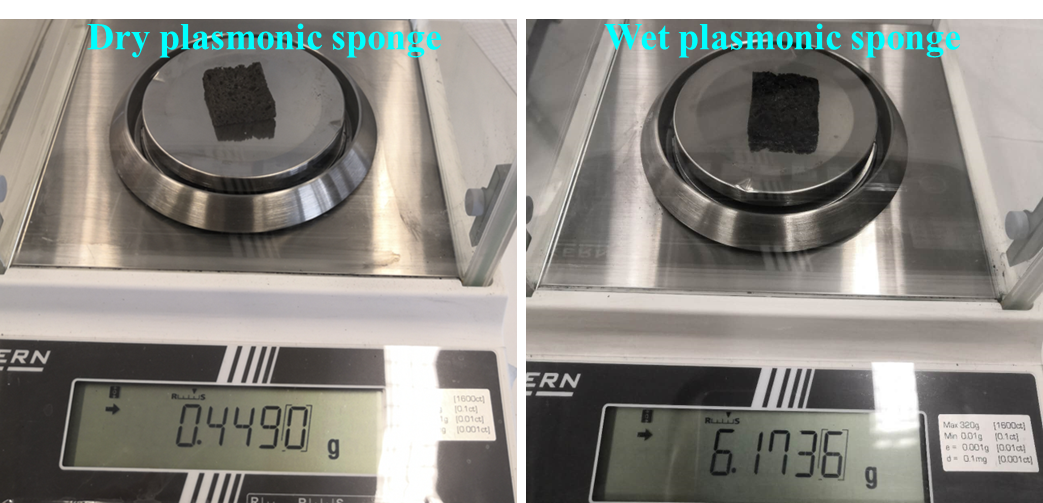


**Figure S7** The weights of plasmonic sponge at dry (at the left) and wet (at the right) state. *m*_dry_ = 0.4490 g and *m*_wet_ = 6.1736 g.


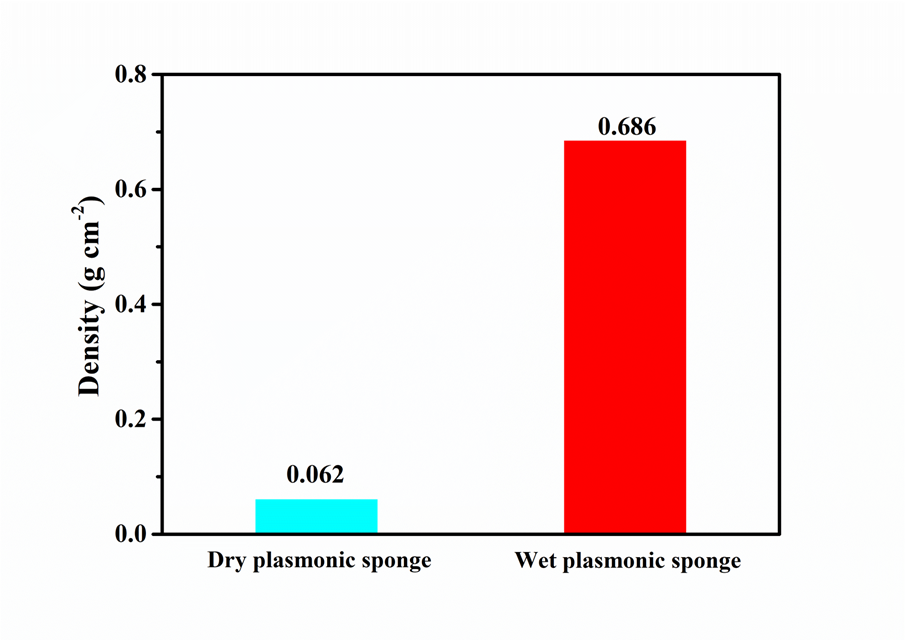


**Figure S8** The densities of plasmonic sponge at dry and wet state.

Calculation details:

Volume of dry plasmonic sponge: 2.7 × 2.7 × 1 = 7.29 cm^3^

Density of dry plasmonic sponge: 0.4490 ÷ 7.29 = 0.062 g·cm^-3^

Volume of wet plasmonic sponge: 3.0 × 3.0 × 1 = 9 cm^3^

Density of wet plasmonic sponge: 6.1736 ÷ 9 = 0.686 g·cm^-3^


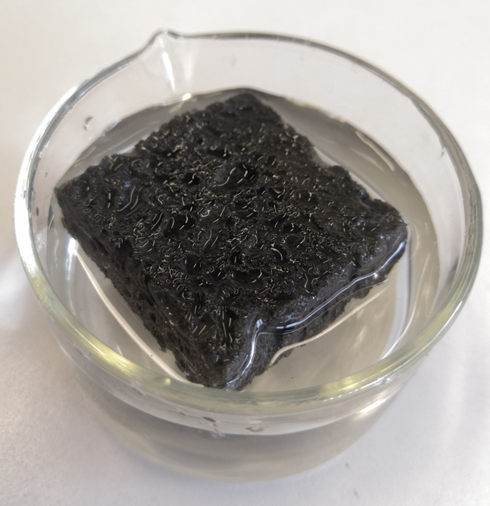


**Figure S9** Photograph of plasmonic sponge floating on water.


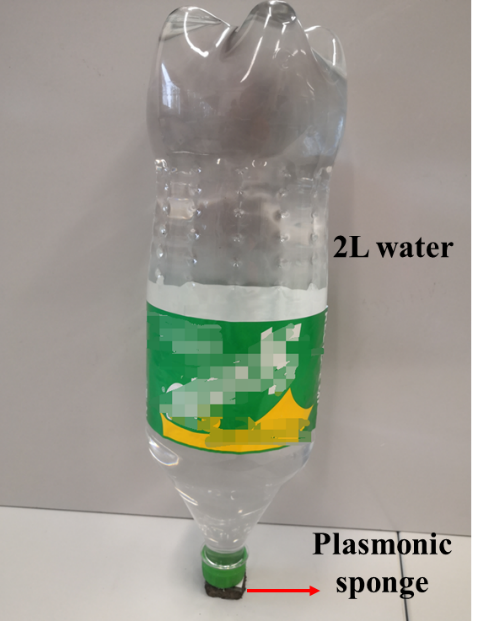


**Figure S10** Plasmonic sponge can withstand ~ 20 N water without being destroyed and can return back to the initial state after stress release, demonstrating its good elastic property.


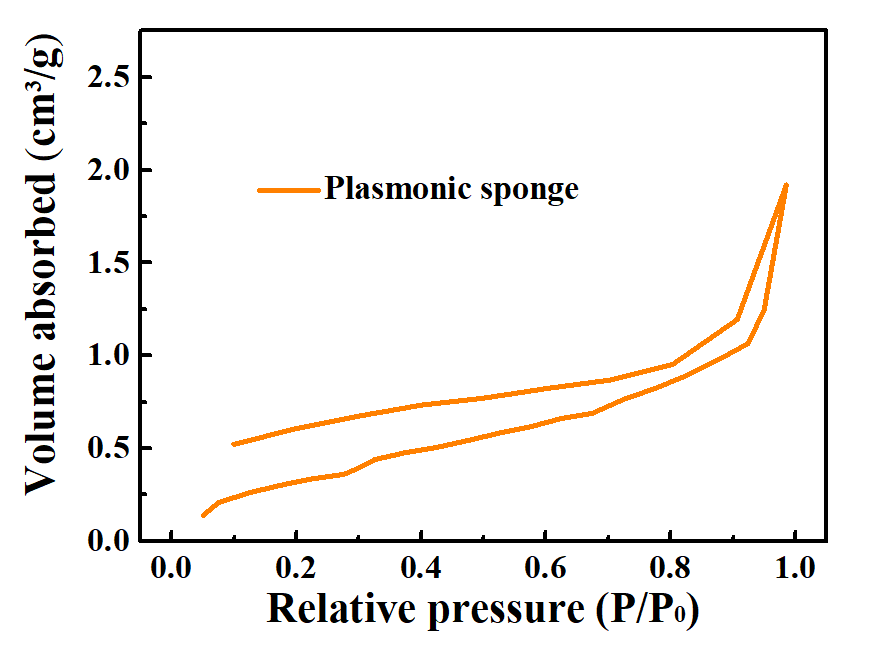


**Figure S11** BET data of the plasmonic sponge. According to the result, the specific surface area of the plasmonic sponge is 1.312 m^2^/g.

The maximum evaporation area = the specific surface area of the absorber × the mass of absorber = 1.312 m^2^/g × 0.449 g = 5891 cm^2^

**Figure S12** Experimental reflection spectrum of both pristine and plasmonic sponges in different media.


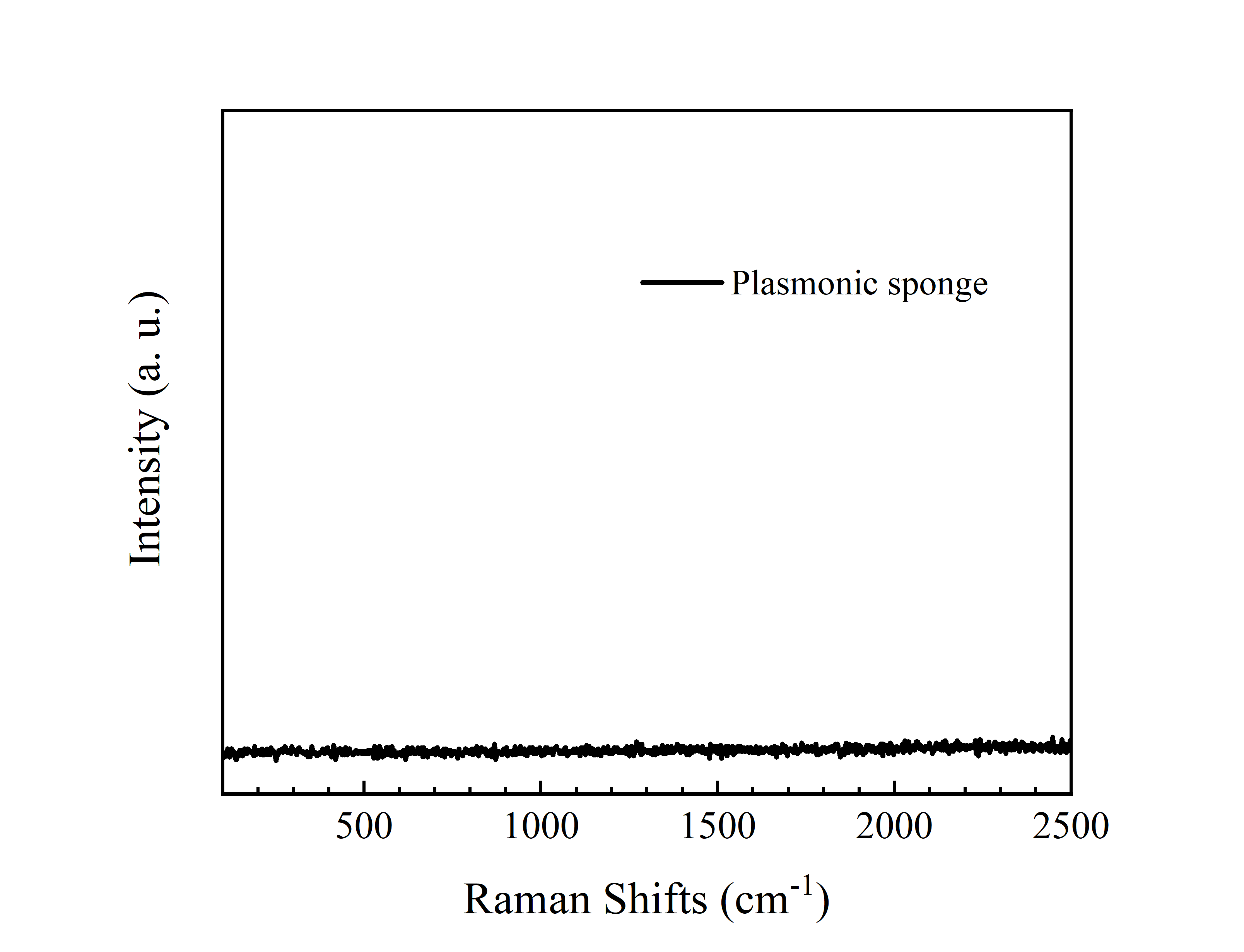


**Figure S13** Raman spectra of plasmonic sponge


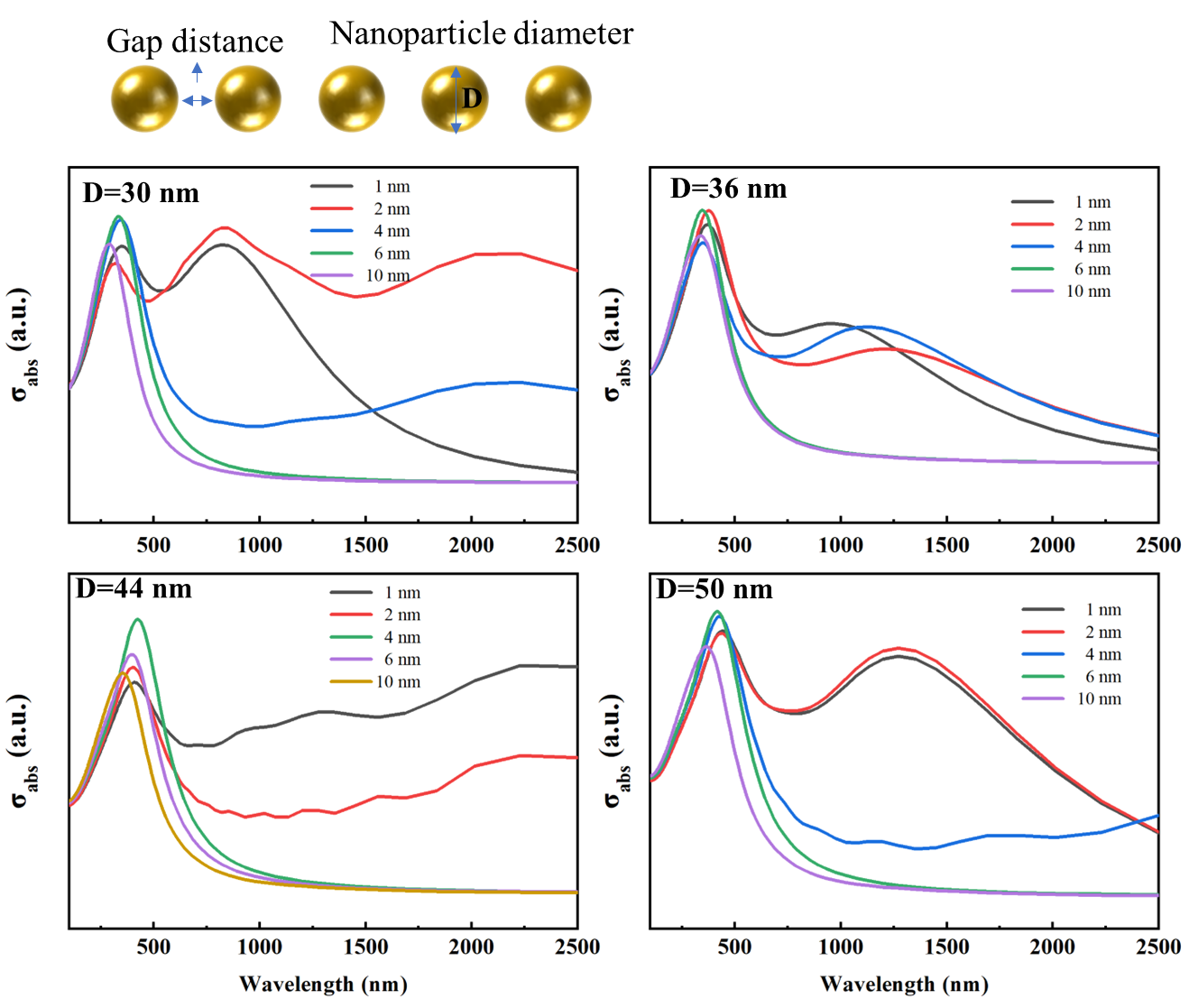


**Figure S14** Nanoparticle size- and inter-particle gap distance-dependent absorption cross.


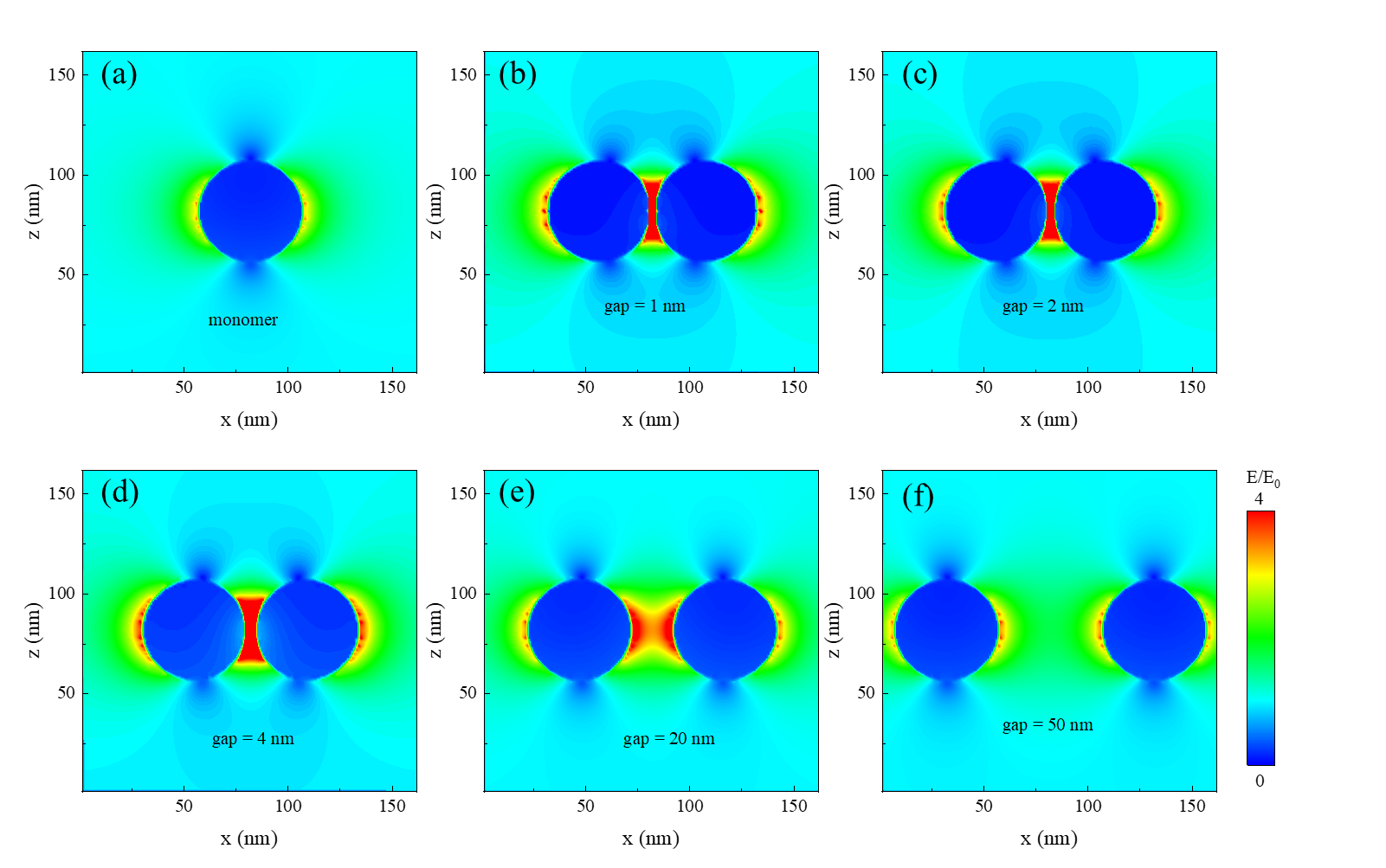


**Figure S15** FDTD-calculated electric field distributions of (a) an isolated single Pd nanoparticle, (b) two Pd nanoparticles with a distance of 1 nm, (c) 2 nm, (d) 4 nm, (e) 20 nm, and (f) 50 nm. The diameter of Pd nanoparticles is~50 nm. The incident wavelength is 520 nm.


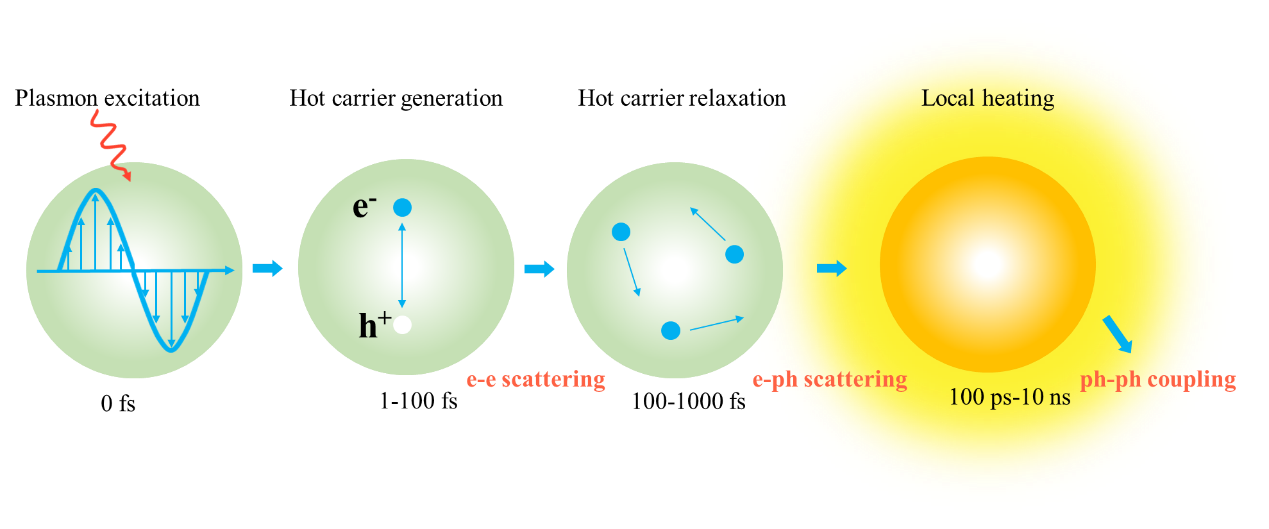


**Figure S16** Mechanism of local heating in a Pd nanoparticle.


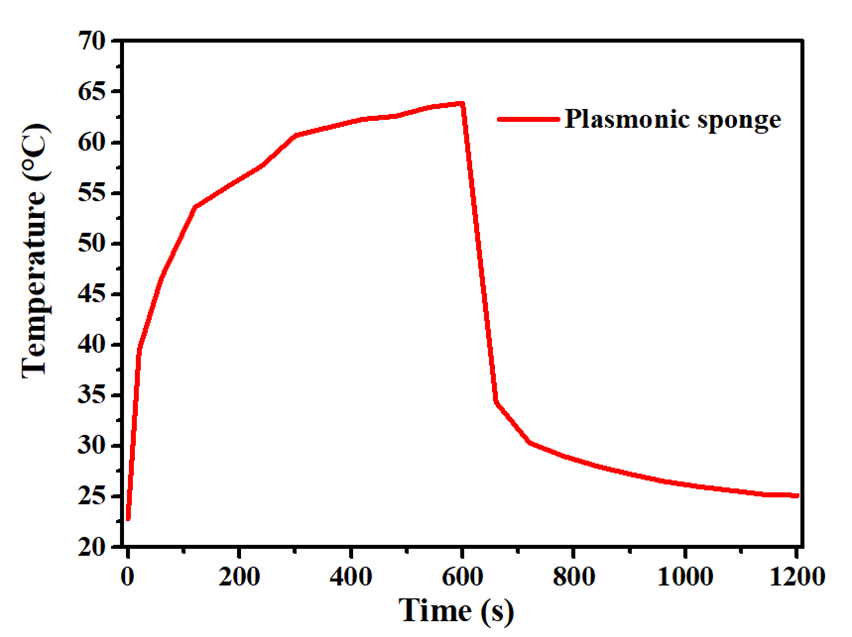


**Figure S17** Photothermal curve of plasmonic sponge under one sun irradiation.


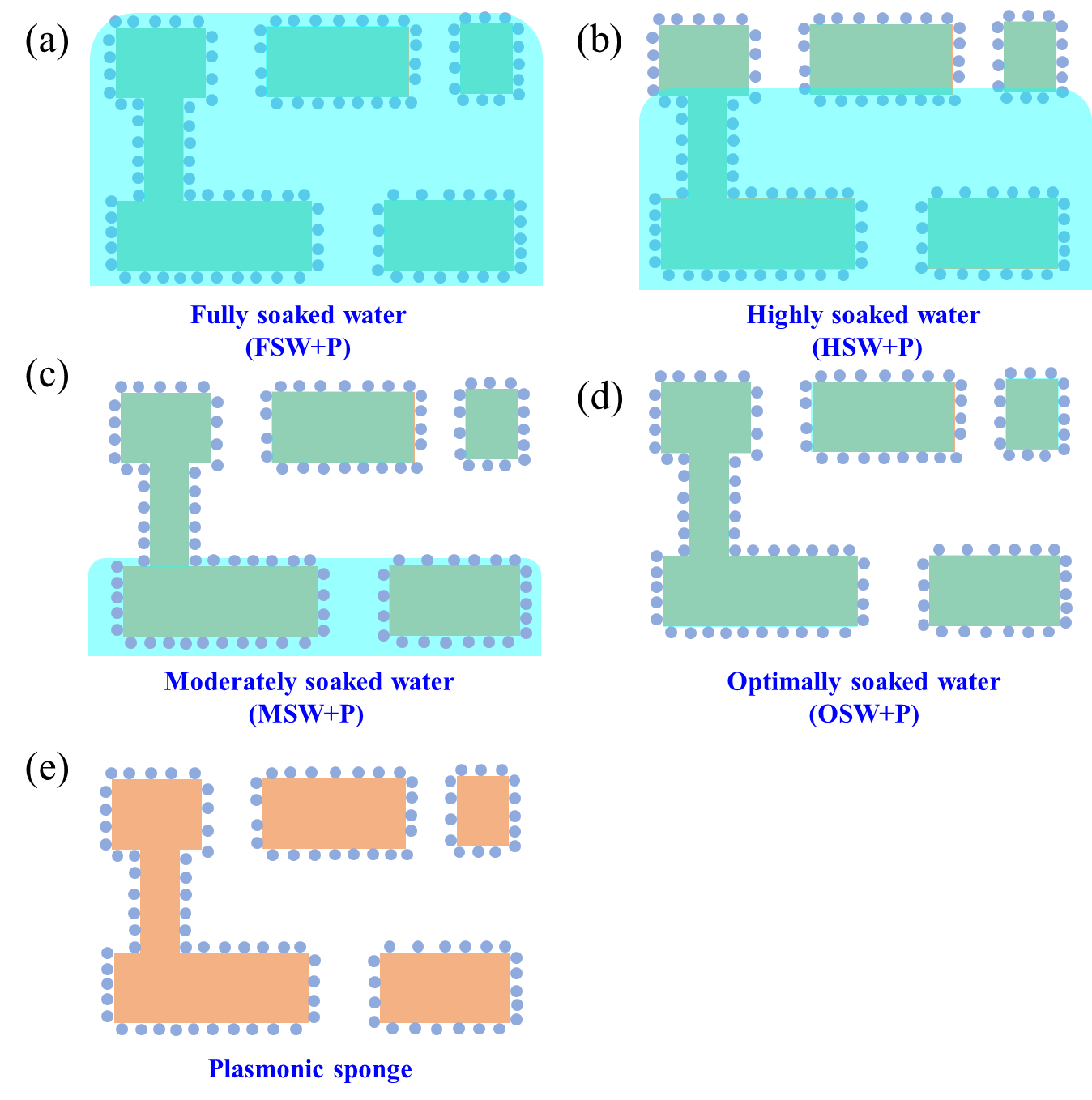


**Figure S18** Schematic illustrations for (a) fully soaked water, (b) highly soaked water, (c) moderate soaked water and (d) optimally soaked water in plasmonic sponges. (e) Pristine plasmonic sponge without any soaked water for reference.


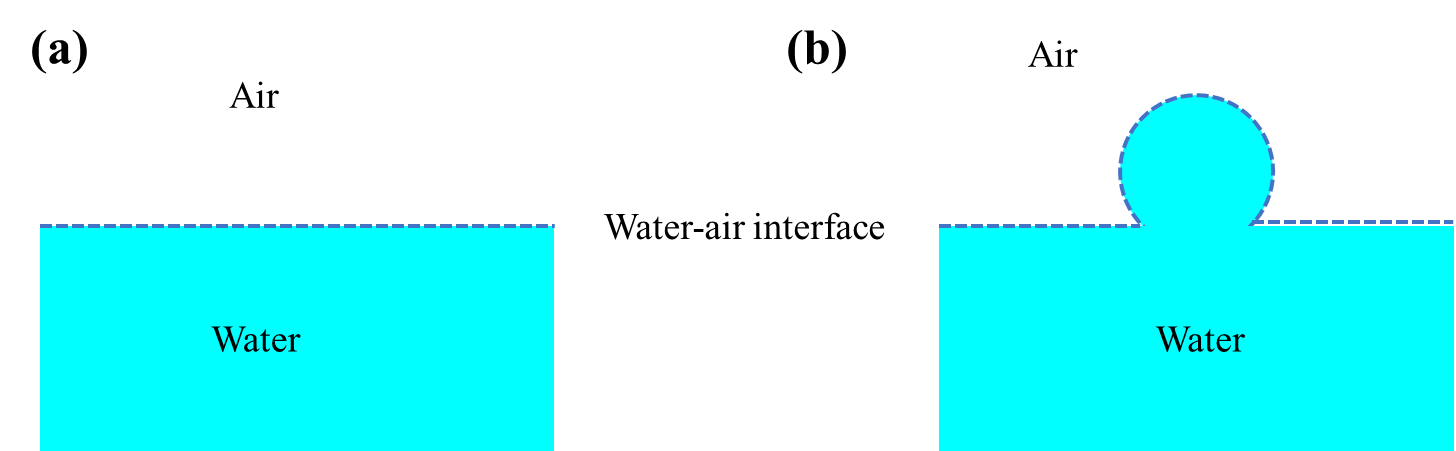


**Figure S19** (a) Schematic illustration of flat water-air interface, and (b) nanostructured water-air interface.


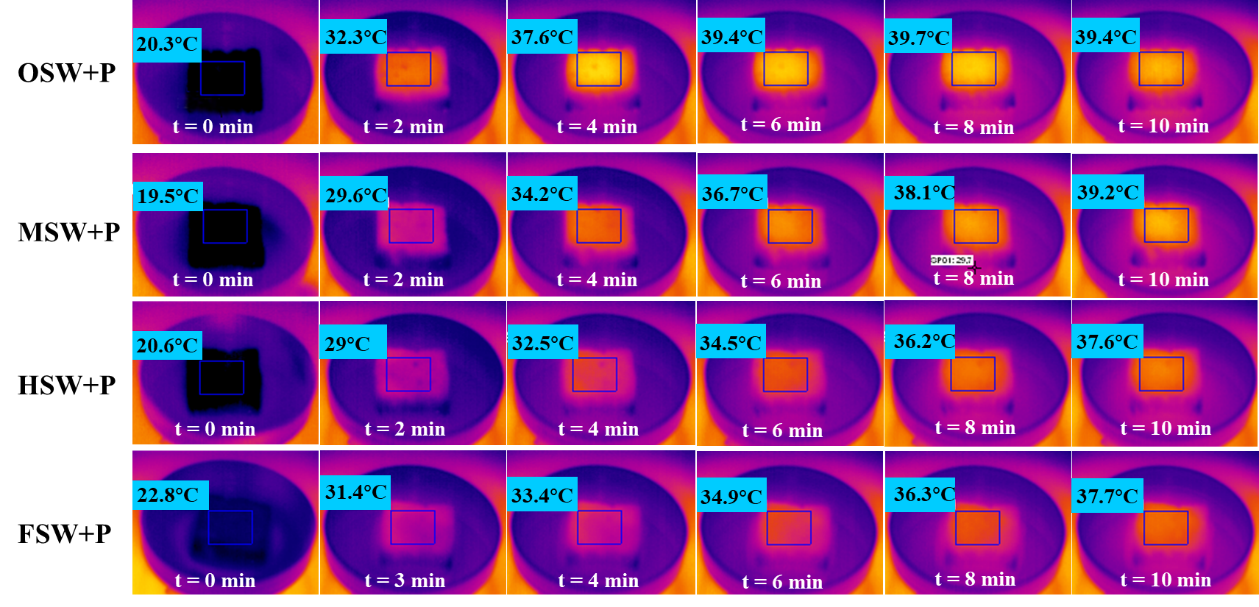


**Figure S20** Photothermal image of the different amounts of soaked water in plasmonic sponges under illumination with one sun after different times.

**Figure S21** The XRD results for the initial sample and after 24 times photothermal experiments. Almost no differences can be observed, showing its excellent stability.


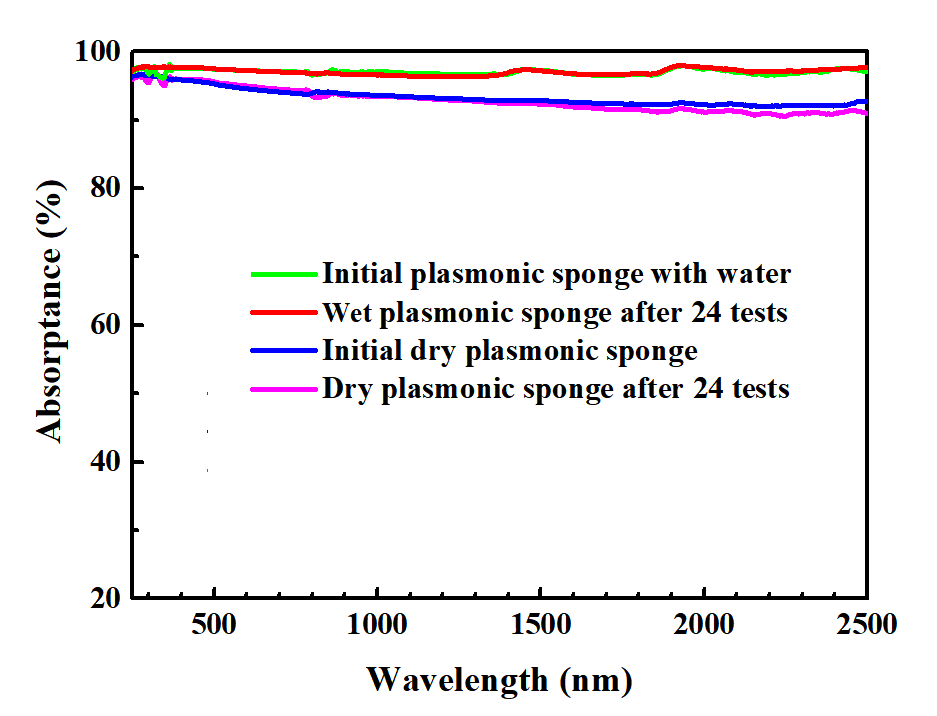


**Figure S22** The absorptance results for the initial sample and after repeating 24 photothermal experiments.


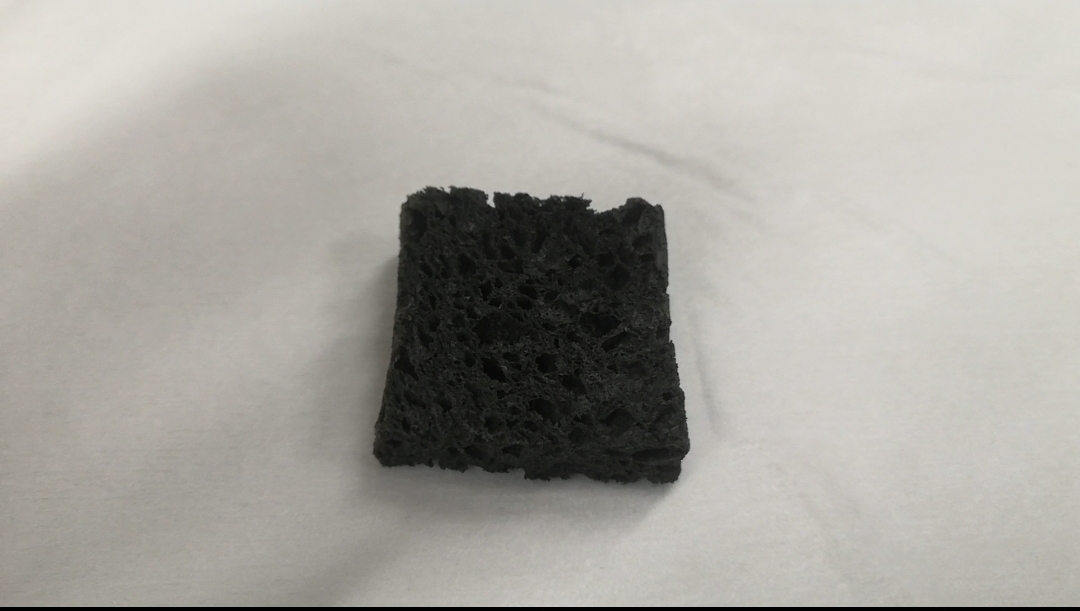


**Figure S23** The photograph of plasmonic sponge after repeating 24 photothermal cycling experiments.


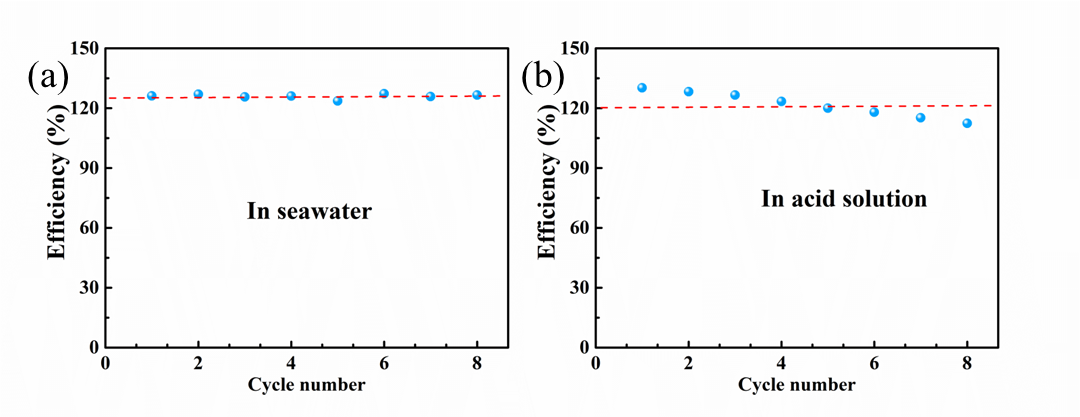


**Figure S24** Stability tests for the plasmonic sponge in seawater (a) and in acid solution (b).


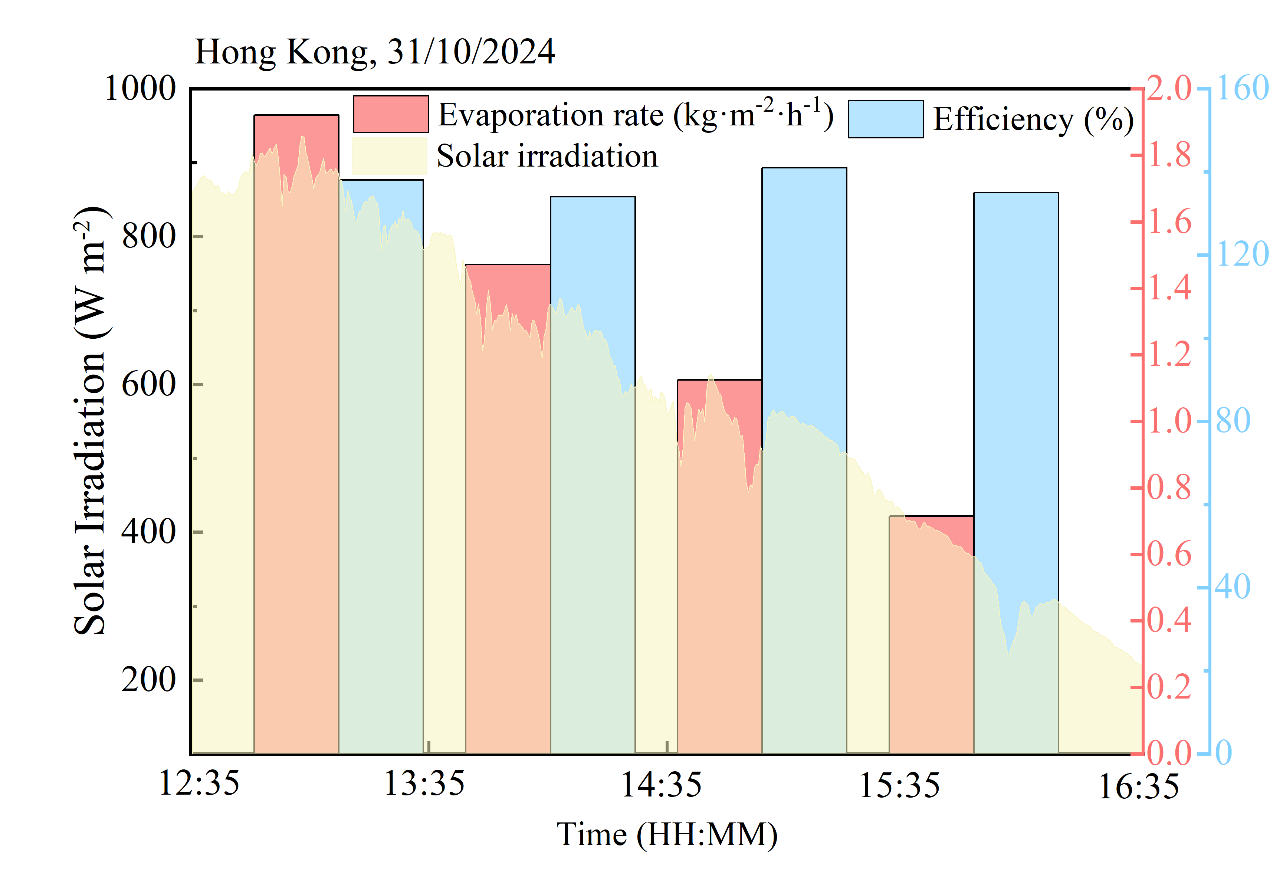


**Figure S25** Evaporation rate and solar-to-vapor efficiency of the plasmonic sponge (OSW+P) at real outdoor experiments (taking place in Hong Kong at October 31, 2024). The light yellow color, pink color and light blue color represent solar irradiation, evaporation rate and solar-to-vapor efficiency.


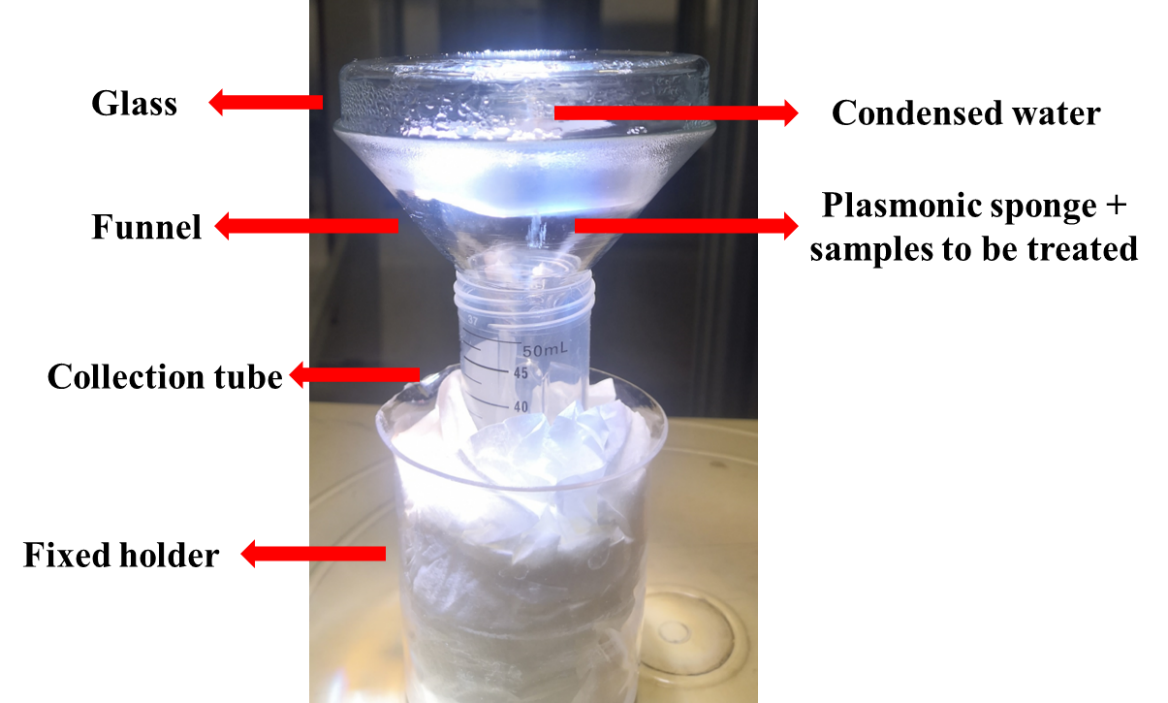


**Figure S26** Purified water collection experimental set-up. Samples, such as metal ion solutions, MO solution and seawater, are placed in a plastic container and plasmonic sponge is placed in the liquid. The glass is used to produce condensed water on its wall under the irradiation. Finally, the condensed water will flow from the glass and funnel into a collection tube.


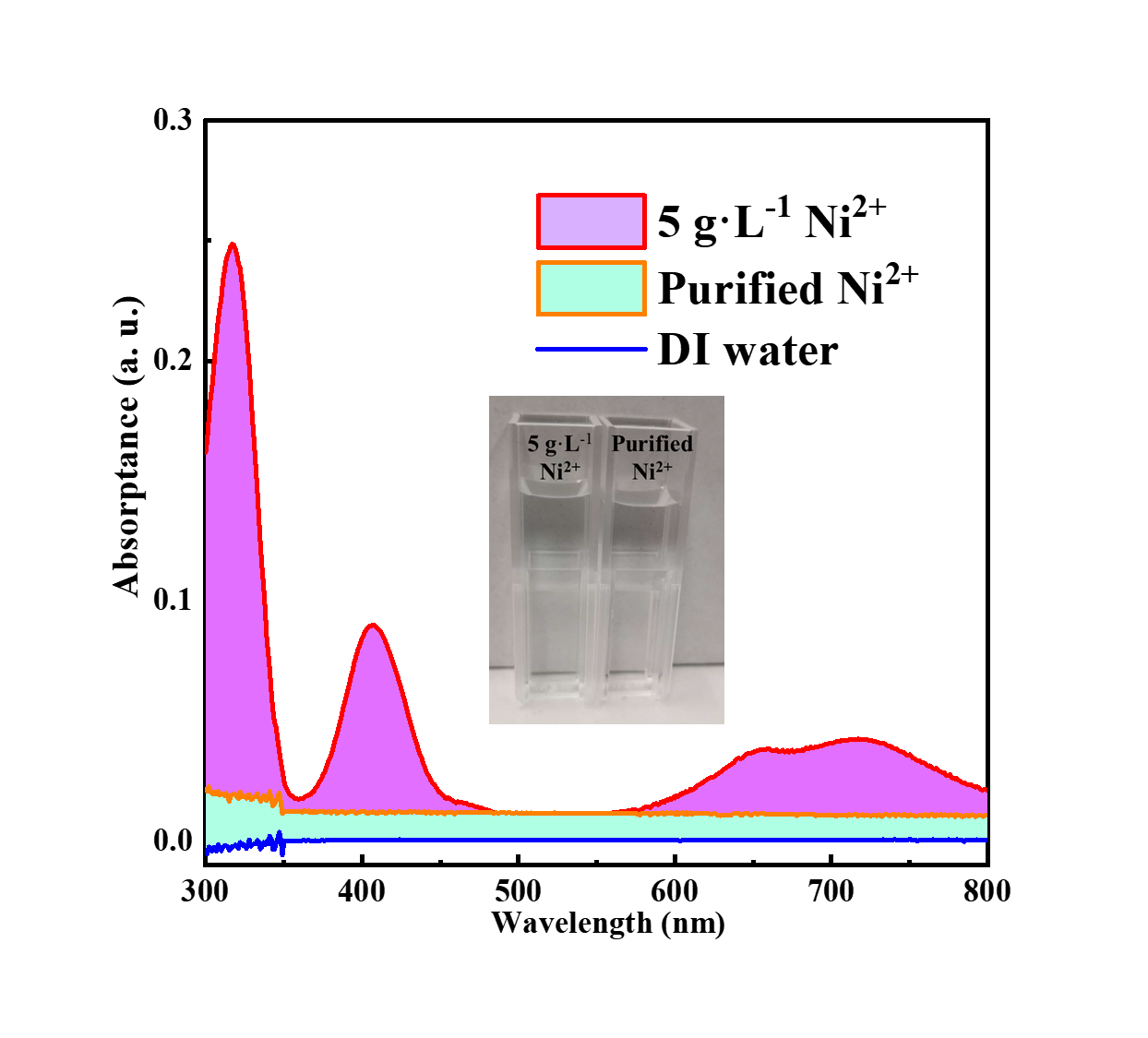


**Figure S27** UV-Vis absorption spectra of Ni^2+^solutions before and after purification by the plasmonic sponge. The insets are the corresponding photographs before and after purification.


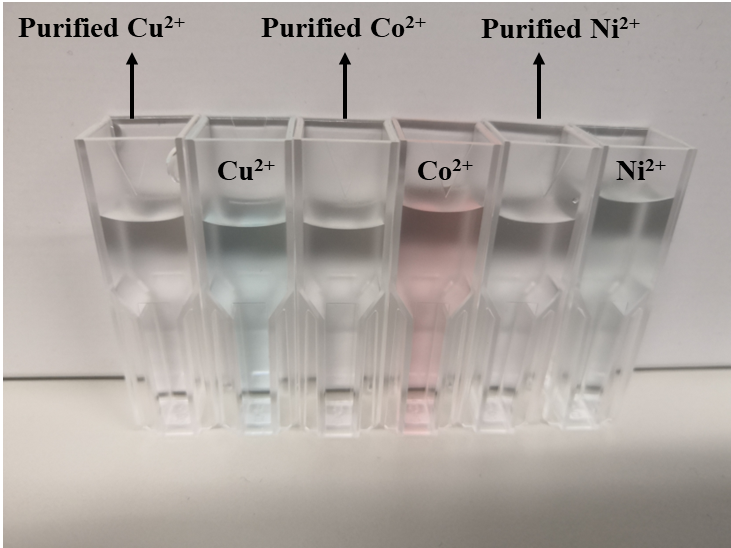


**Figure S28** Photographs of Cu^2+^, Co^2+^ and Ni^2+^ solution before and after purification. From the left to the right, they are purified Cu^2+^ solution, 5 g·L^-1^ CuSO_4_ solution, purified Co^2+^ solution, 5 g·L^-1^ CoCl_2_ solution, purified Ni^2+^ solution, 5 g·L^-1^ NiCl_2_ solution, respectively, corresponding transparent color, light blue color, transparent color, light pink color, transparent color, light green color.

**Figure S29** The concentration of Cu^+^, Co^2+^, and Ni^2+^ before and after purification.

**Figure S30** Measured concentrations of Na^+^, Mg^2+^, and Ca^2+^ in purified seawater before and after desalination.


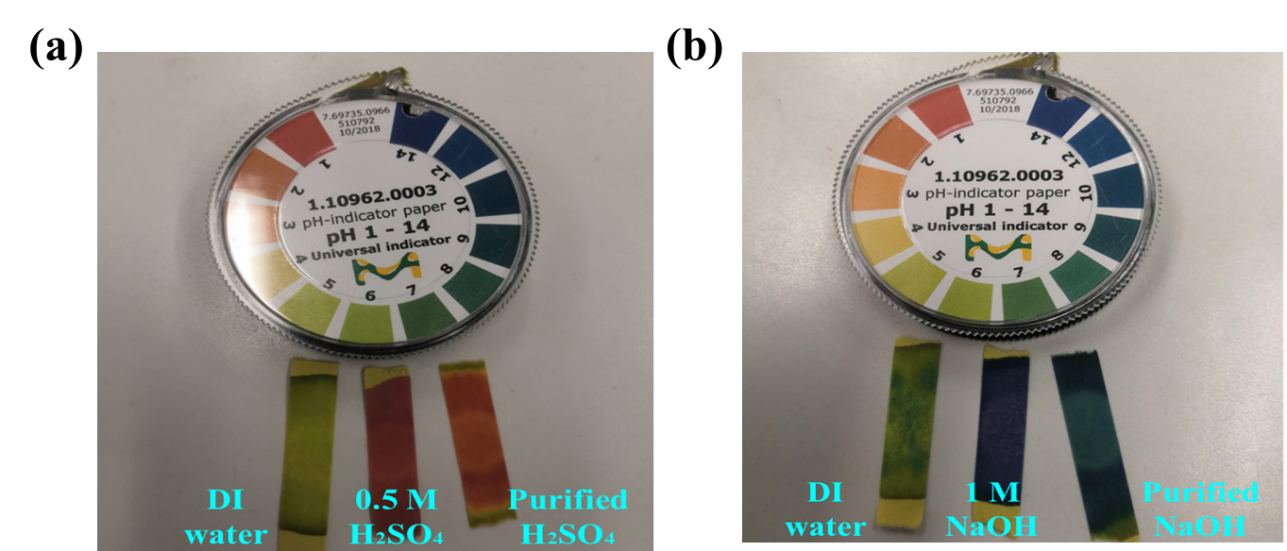


**Figure S31** Photographs of DI water, 0.5 M H_2_SO_4_ and 1 M KOH before (a) and after (b) purification by the plasmonic sponge.

**Table S1** The cost for raw materials of four typical plasmonic metal nanostructures, such as Al/AAO (Ref.10 in the main text), nanoporous Au (Ref.7 in the main text) and Au/AAO, for comparison with plasmonic sponge. They are compared with a similar thickness.

| Materials | Raw materials | Quantity | Price (US$)^a^ |
| --- | --- | --- | --- |
| Al/AAO | Al, AAO | ~, 1 inch | ~ 65^b^ |
| Nanoporous Au | Au, Cu, HNO_3_ | 8 mm^3^ (Cu_99_Au_1_), 100 mL (70 wt. %) | ~70 |
| Au/AAO | Au, AAO | 85 nm, 1 inch | ~150^c^ |
| Pd/sponge | PdCl_2_, ethanol, sponge | 14 mg, 2.5 mL, 7.29 cm^2^ | ~1.8 |

^a^ The prices of chemicals of Al, Cu HNO_3_, PdCl_2_ are from Sigma-Aldrich company. ^b & c^ The price of Al and Au are from the cost of PVD deposition and AAO is from the Whatman company.

**Table S2** Solar evaporation performance of the plasmonic sponge compared with the latest 2D and 3D interfacial evaporation systems.

| Materials | Evaporation rate  (kg·m^-2^·h^-1^) | Efficiency | Design | Ref. |
| --- | --- | --- | --- | --- |
| Janus CNT membrane | 1.59 | 107.4% | 2D | ^[1]^ |
| carbon black nanoparticles/cellulose | 1.62 | 110% | 3D | ^[2]^ |
| 3D Carbon nanotubes | 1.47 | 98.9% | 3D | ^[3]^ |
| NiPS_3_/sponge | 1.48 | 93.5% | 2D | ^[4]^ |
| HNG^a^ | 3.2* | 94% | 3D | ^[5]^ |
| Activated carbon | 25.3 | - | 3D | ^[6]^ |
| Pd/SnS_2_/sponge | 1.72 | 89.2% | 2D | ^[7]^ |
| MXene/Au@Cu_2−x_S | 2.02 | 96.1% | 2D | ^[8]^ |
| MoS_2_/sponge | 1.204 | 86.2% | 2D | ^[9]^ |
| MoS_2_ hydrogel | 3.297* | 93.4% | 2D | ^[10]^ |
| **Pd NPs/sponge** | **2.022** | **131%** | **3D** | **This work** |

a: HNG: a hierarchically nanostructured gel based on polyvinyl alcohol and polypyrrole.

*: For hydrogel absorber systems: The solar-to-vapor efficiency is calculated using a significantly decreased Δ*H* (the evaporation enthalpy of water). ^[11–15]^

References

[1] Q. Xia, Y. Pan, B. Liu, X. Zhang, E. Li, T. Shen, S. Li, N. Xu, J. Ding, C. Wang, C. D. Vecitis, G. Gao, *Sci. Adv.* **2024**, *10*, eadj3760.

[2] X. Li, J. Li, J. Lu, N. Xu, C. Chen, X. Min, B. Zhu, H. Li, L. Zhou, S. Zhu, T. Zhang, J. Zhu, *Joule* **2018**, *2*, 1331.

[3] L. Wu, Z. Dong, Z. Cai, T. Ganapathy, N. X. Fang, C. Li, C. Yu, Y. Zhang, Y. Song, *Nat. Commun.* **2020**, *11*, 521.

[4] H. Wang, Y. Bo, M. Klingenhof, J. Peng, D. Wang, B. Wu, J. Pezoldt, P. Cheng, A. Knauer, W. Hua, H. Wang, P. A. van Aken, Z. Sofer, P. Strasser, D. M. Guldi, P. Schaaf, *Adv. Funct. Mater.* **2024**, *34*, 2310942.

[5] F. Zhao, X. Zhou, Y. Shi, X. Qian, M. Alexander, X. Zhao, S. Mendez, R. Yang, L. Qu, G. Yu, *Nat. Nanotechnol.* **2018**, *13*, 489.

[6] M. Zhang, N. Hu, Y. Guo, W. Wu, L. Fan, D. Lin, J. Wang, K. Yang, *Adv. Sci.* **2024**, *11*, 2402583.

[7] P. Cheng, H. Wang, H. Wang, D. Wang, P. A. van Aken, P. Schaaf, *Small* **2024**, *n/a*, 2400588.

[8] H.-S. Kang, J.-W. Zou, Y. Liu, L. Ma, J.-R. Feng, Z.-Y. Yu, X.-B. Chen, S.-J. Ding, L. Zhou, Q.-Q. Wang, *Adv. Funct. Mater.* **2023**, *33*, 2303911.

[9] Q. Wang, F. Jia, A. Huang, Y. Qin, S. Song, Y. Li, M. A. C. Arroyo, *Desalination* **2020**, *481*, 114359.

[10] P. Liu, Y. Hu, X.-Y. Li, L. Xu, C. Chen, B. Yuan, M.-L. Fu, *Angew. Chem., Int. Ed.* **2022**, *61*, e202208587.

[11] A. Taranova, E. Moretti, K. Akbar, G. Dastgeer, A. Vomiero, *Nano Energy* **2024**, *128*, 109872.

[12] Z. Yu, R. Gu, Y. Tian, P. Xie, B. Jin, S. Cheng, *Adv. Funct. Mater.* **2022**, *32*, 2108586.

[13] X. Liu, D. D. Mishra, X. Wang, H. Peng, C. Hu, *J. Mater. Chem. A* **2020**, *8*, 17907.

[14] C. Dang, Y. Cao, H. Nie, W. Lang, J. Zhang, G. Xu, M. Zhu, *Nat. Water* **2024**, *2*, 115.

[15] X. Mu, Y. Gu, P. Wang, A. Wei, Y. Tian, J. Zhou, Y. Chen, J. Zhang, Z. Sun, J. Liu, L. Sun, S. Tanemura, L. Miao, *Solar Energy Materials and Solar Cells* **2021**, *220*, 110842.
